# Alkyl Chloride-Functionalized Polymers Mediate Oxidation of Thioethers Initiated by Ionizing Radiation

**DOI:** 10.1021/acsapm.5c00054

**Published:** 2025-03-19

**Authors:** Juncheng Liu, Irene Piergentili, Bing Xu, Antonia G. Denkova, Rienk Eelkema

**Affiliations:** † Department of Chemical Engineering, Delft University of Technology, van der Maasweg 9, 2629 HZ Delft, Netherlands; ‡ Department of Radiation Science and Technology, Delft University of Technology, Mekelweg 15, 2629 JB Delft, Netherlands

**Keywords:** ionizing radiation, reactive oxygen species, micelles, chlorinated polymers, triggered release

## Abstract

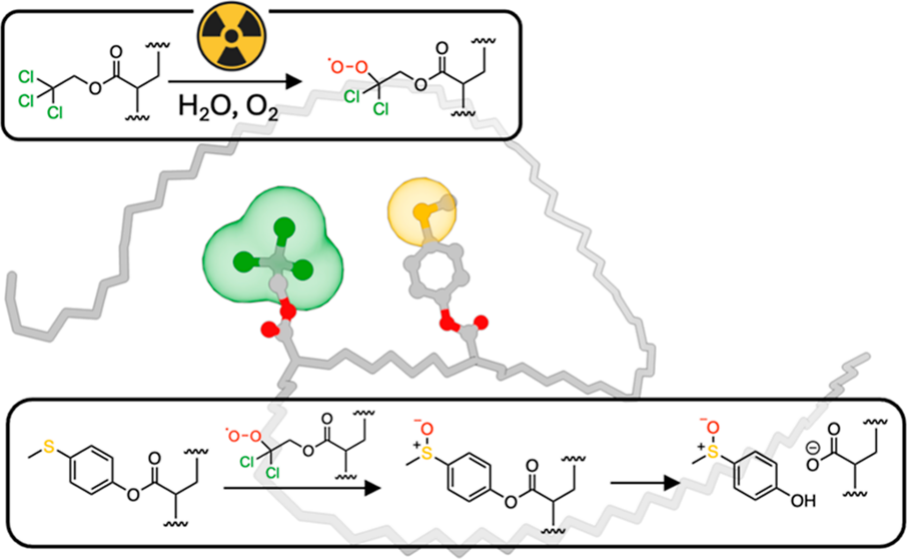

Irradiation of aqueous solutions containing alkyl chlorides generates peroxyl radicals by reactions of alkyl chlorides, aqueous electrons, and dissolved oxygen. The peroxyl radical can oxidize thioethers to sulfoxides, a transformation that has relevance for targeted or triggered drug delivery. However, small-molecule alkyl chlorides can induce liver damage, which limits their potential for application in anticancer therapy. Here, we show that alkyl chlorides bound to a hydrophilic random copolymer chain behave similar to small-molecule alkyl chlorides. Our work shows that using polymeric alkyl chlorides can be an alternative to small-molecule alkyl chlorides provided that the alkyl chloride functionalities are easily accessible to aqueous electrons.

## 1 Introduction

Radiotherapy and chemotherapy are two primary cancer treatments used in clinical practice.^1^ Radiotherapy uses ionizing radiation to damage cancerous cells, while chemotherapy employs antitumor drugs to kill cells or inhibit their proliferation. However, both ionizing radiation and antitumor drugs can also harm healthy tissues during treatment. The effectiveness of these therapies is determined by the balance between tumor damage and systemic toxicity to normal tissues. Ionizing radiation-induced drug release presents a promising strategy to reduce side effects when combining radiotherapy with chemotherapy.^2−4^ As water is the most abundant matter in tissues, radiation mostly deposits its energy to water, causing water radiolysis.^5^ The drug release process is then mediated by reactive species generated from water radiolysis. Examples of such systems are polymeric nanocarriers encapsulating drugs to minimize systemic toxicity, which upon exposure to ionizing radiation can degrade, releasing the drug precisely at the site of irradiation.^6−9^ This approach profits from the precise spatial control of radiotherapy to ensure targeted drug release exclusively at the tumor site. Although several chemical reactions have been explored to achieve effective radiation-induced drug release,^10−14^ more efficient reactions must be developed to apply this strategy in clinical settings.

**Figure 1:**
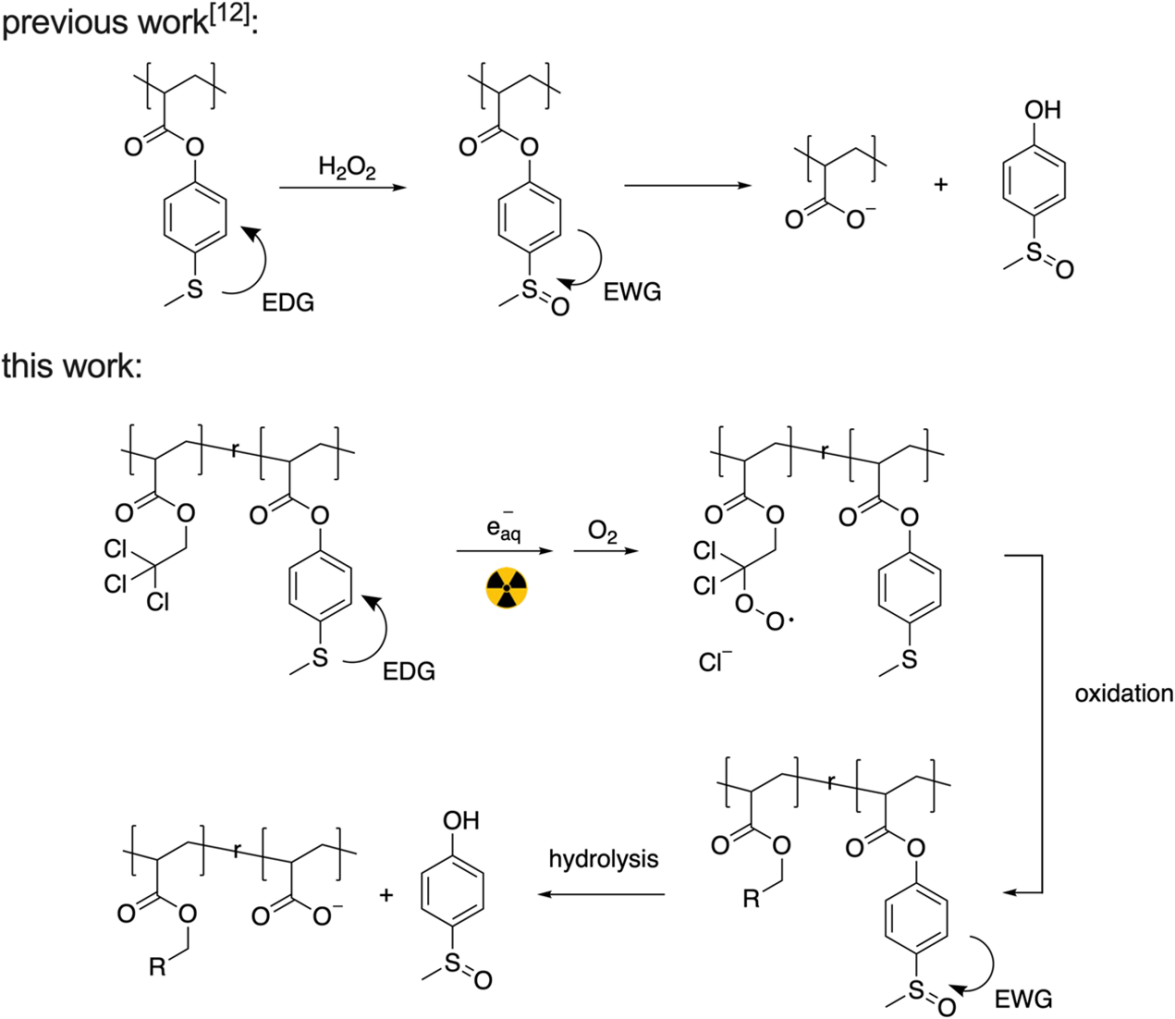
Schematic illustration of radiation-induced oxidation from an electron-donating (EDG) thioether to electron-withdrawing sulfoxide.

Organic compounds containing thioethers exhibit distinctive properties after the oxidation of the sulfur atom. The thioether group itself is hydrophobic, whereas its oxidation products, sulfoxides and sulfones, are hydrophilic.^15^ Exploiting this property, Napoli et al.^16^ developed an ABA-type block copolymer, using poly(propylenesulfide) as the hydrophobic block. Oxidation of the thioether group switched the hydrophobic block to a hydrophilic one, leading to vesicle disassembly. For aromatic thioethers, an intriguing property is the change in electronic effects upon oxidation.^17^ While the thioether group is electron-donating, its oxidized forms, sulfoxide and sulfone, are electron-withdrawing groups.^18^ Phenyl acetate esters with electron-withdrawing substituents on the aromatic ring are more labile toward hydrolysis than those with electron-donating groups. Our research group applied this principle to design H_2_O_2_-responsive amphiphilic block copolymers (see Figure 1),^19^ whereas poly(4-(methylthio)phenyl acrylate) (MTPA) functions as the hydrophobic block. Upon H_2_O_2_ addition, MTPA undergoes oxidation followed by hydrolysis, converting the hydrophobic core into a hydrophilic polyacrylate polyanion, which leads to micelle disassembly. However, the oxidation of thioethers by H_2_O_2_ is slow, requiring several days to weeks to respond to cellular H_2_O_2_ concentrations (50–100 μM). While enzymatic and organic catalysts have been developed to accelerate this process, controlling catalyst concentration in cellular environments poses challenges.^20,21^ Thus, more efficient oxidants are needed to make this approach viable for biological systems.

In previous work, we demonstrated the oxidative cleavage of a stilbene derivative, induced by ionizing radiation.^22^ The presence of 0.1 vol % of an alkyl chloride such as chloroform or trichloroethanol in aqueous solution significantly enhances the stilbene oxidation products’ yield. We hypothesized that the aqueous electrons generated from water radiolysis can react rapidly with the alkyl chloride to form a carbon-centered radical and a chloride anion. In the presence of oxygen, the carbon-centered radical reacts with molecular oxygen and forms a strong oxidant, i.e., peroxyl radical. In the current work, we show that a thioether, dissolved in aqueous solutions with alkyl chlorides, can be oxidized to a sulfoxide when it is exposed to ionizing radiation. We use this reaction to trigger the hydrophilicity switch of a reactive oxygen species (ROS)-responsive block copolymer aggregate (Figure 1). Although oxidation and hydrolysis are observed, the amount of polyacrylate anion is not enough to induce aggregate disassembly after exposure to 600 Gy gamma-radiation. We copolymerized alkyl chloride and MTPA to reduce the potential toxicity of small-molecule alkyl chlorides.^23^ However, we find that when the alkyl chloride is incorporated in the hydrophobic block, the reaction of alkyl chloride and aqueous electrons is inhibited, leading to reduced yields of the oxidation products. We anticipated that the hydrophilic nature of the hydrated electron bars it from entering the hydrophobic core of micellar aggregates, leading to a reduced reactivity. To test this hypothesis, we synthesized a random-copolymer rP2 using MTPA, alkyl chloride-acrylate, and *N*,*N*-dimethylacrylamide as monomers. rP2 is fully soluble in aqueous solvent, and the alkyl chloride-based monomer is solvated. rP2 solutions show higher yields of oxidized product after irradiation than its aggregate forming analogues. These findings show that the alkyl chloride covalently bound to a polymer can show similar reactivity compared to small-molecule alkyl chlorides, which paves the way to the future application of alkyl chloride-mediated oxidation induced by ionizing radiation.

## 2 Experimental Section

### 2.1 Materials and Instruments

All compounds were purchased from commercial suppliers (Sigma-Aldrich, Tokyo Chemical Industry, and abcr Gute Chemie) and used without further purification unless otherwise specified. THF was distilled to remove the radical inhibitor. Reactions were monitored by thin-layer chromatography on a silica gel plate and visualized by ultraviolet (UV) light (254 nm) or stained using a KMnO_4_/OH^–^ solution. Flash column chromatography was carried out on a 30 cm column loaded with 230–400 mesh silica gel. ^1^H NMR spectra of small molecules were recorded on an Agilent-400 MR DD2 (399.67 MHz) or a Bruker 600 MHz at 298 K. Dynamic light scattering (DLS) was performed on a Zetasizer Pro instrument equipped with a laser operating at 633 nm. Gel permeation chromatography (GPC) was performed on a Prominence-I GPC system (Shimadzu) with a KD804 column (Shodex, 8 mm (i.d.) × 30 cm) and equipped with differential refractive index and UV detectors. DMF with 10 mM LiBr was used as the mobile phase (1 mL/min). Polystyrene standards were used for calibration. Irradiation with gamma rays was performed using a Nordion 220 ^60^Co gamma cell. The dose rate at the experimental date was around 0.1080 Gy/s, which was calculated based on the decay law and the half-life of ^60^Co. The delivered dose was calculated by the dose rate at the date of the experiments multiplied by the exposure time. Radiation was given in one fraction unless otherwise specified.

### 2.2 Synthesis of Polymers

The polymers were synthesized by reversible addition–fragmentation chain transfer (RAFT) polymerization. Specific amounts of RAFT agent, monomers, and *N*,*N*-dimethylformamide (DMF) were added to a Schlenk flask. The solution was bubbled with nitrogen gas for 15 min and then sealed. An ^1^H NMR spectrum at t_0_ was recorded. The reaction mixture was stirred at room temperature in an LED light reactor using 444 nm blue light. The monomer conversion was determined using ^1^H NMR spectroscopy. The product was isolated by first diluting the reaction mixture with DCM (50 mL) and subsequent precipitation (3×) in diethyl ether (500 mL), after which the product was dried in a vacuum oven at 40 °C for 3 days. The degree of polymerization was calculated according to eqs S1–S3.

### 2.3 Preparation and Irradiation of Polymer Solutions

Deuterated phosphate buffer (*d*-PB, 100 mM pD 7.4) was prepared by dissolving 0.28 g of monosodium phosphate monohydrate and 1.13 g of anhydrous disodium phosphate in 100 mL of deuterium oxide (D_2_O). The pD was adjusted by adding 0.1 mg/mL NaOH in D_2_O and calibrated by using a pH meter (HAMILTON, Mettler Toledo).

The polymer solutions were prepared by a solvent switching method. A specific amount of polymer was dissolved in 0.1 mL of THF. The solution was stirred for 10 min followed by slowly adding 1 mL of *d*-PB. The obtained transparent solution was stirred in an open vial for 24 h to evaporate the THF. The solution was transferred to an NMR tube and was exposed to the ^60^Co gamma-irradiator. For DLS measurements, the solution was transferred to a 4 mL glass vial. Polymer solutions in glass vials or NMR tubes were placed in the center of the cell. The samples were irradiated for 555.5 s for 60 Gy and 5555 s for 600 Gy of radiation.

## 3 Results and Discussion

**Figure 2:**
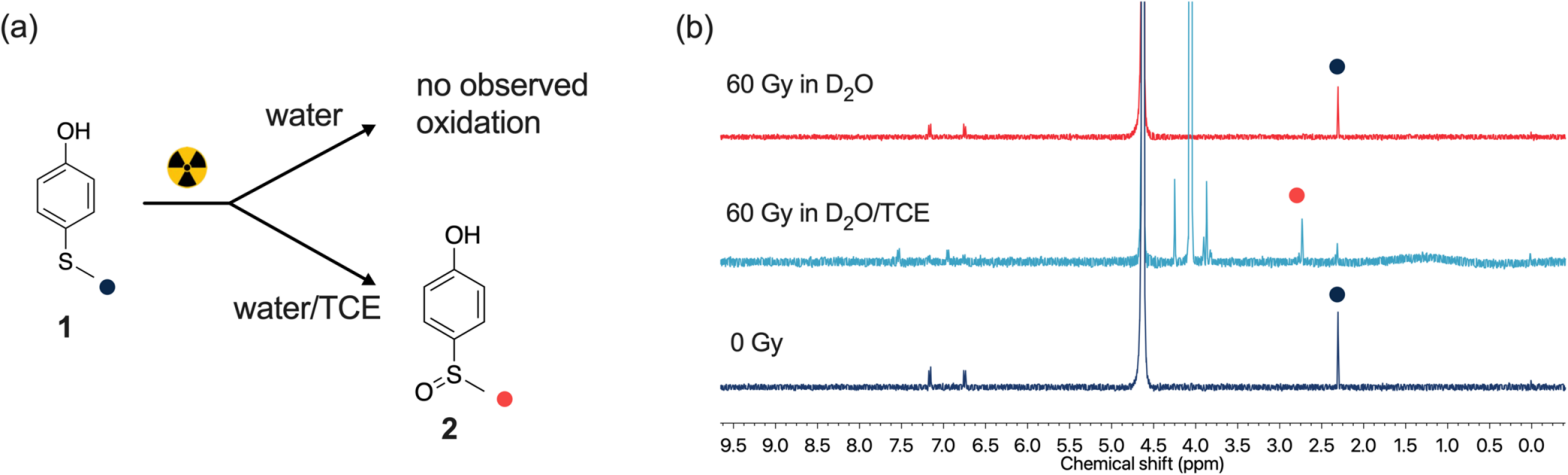
(a) Reactivity of compound **1** under irradiation in water and water/TCE. (b) ^1^H NMR spectrum of compound **1** before and after irradiation (spectra were taken within 3 h after irradiation).

To test the reaction of thioethers under irradiation in water or in water with alkyl chloride, we chose 4-(methylthio)phenol (compound **1**) as the model thioether and 2,2,2-trichloroethan-1-ol (TCE) as the model alkyl chloride. Proton nuclear magnetic resonance spectroscopy (^1^H NMR) was employed to quantify the amount of oxidation. After exposure to γ radiation (60 Gy) in water, the spectrum of the solution of compound **1** (50 μM) remained the same as it was before irradiation (Figure 2b). Although water radiolysis generates hydrogen peroxide, which can oxidize the thioether, we could not detect any oxidation products after irradiation. This is likely caused by the low concentration of H_2_O_2_ generated by 60 Gy (ca. 4.2 μM) and the slow reaction kinetics of thioether oxidation. However, when 0.1 vol % TCE was present during the irradiation, a new methyl peak appeared at 2.73 ppm, corresponding to the formation of sulfoxide compound **2**,^19^ and the conversion of compound **2** remained unchanged following 3 h of incubation (Figure S2). This indicates that the peroxyl radical generated from the water/TCE system can oxidize the thioether, and the oxidation does not proceed when the irradiation is stopped.

**Figure 3:**
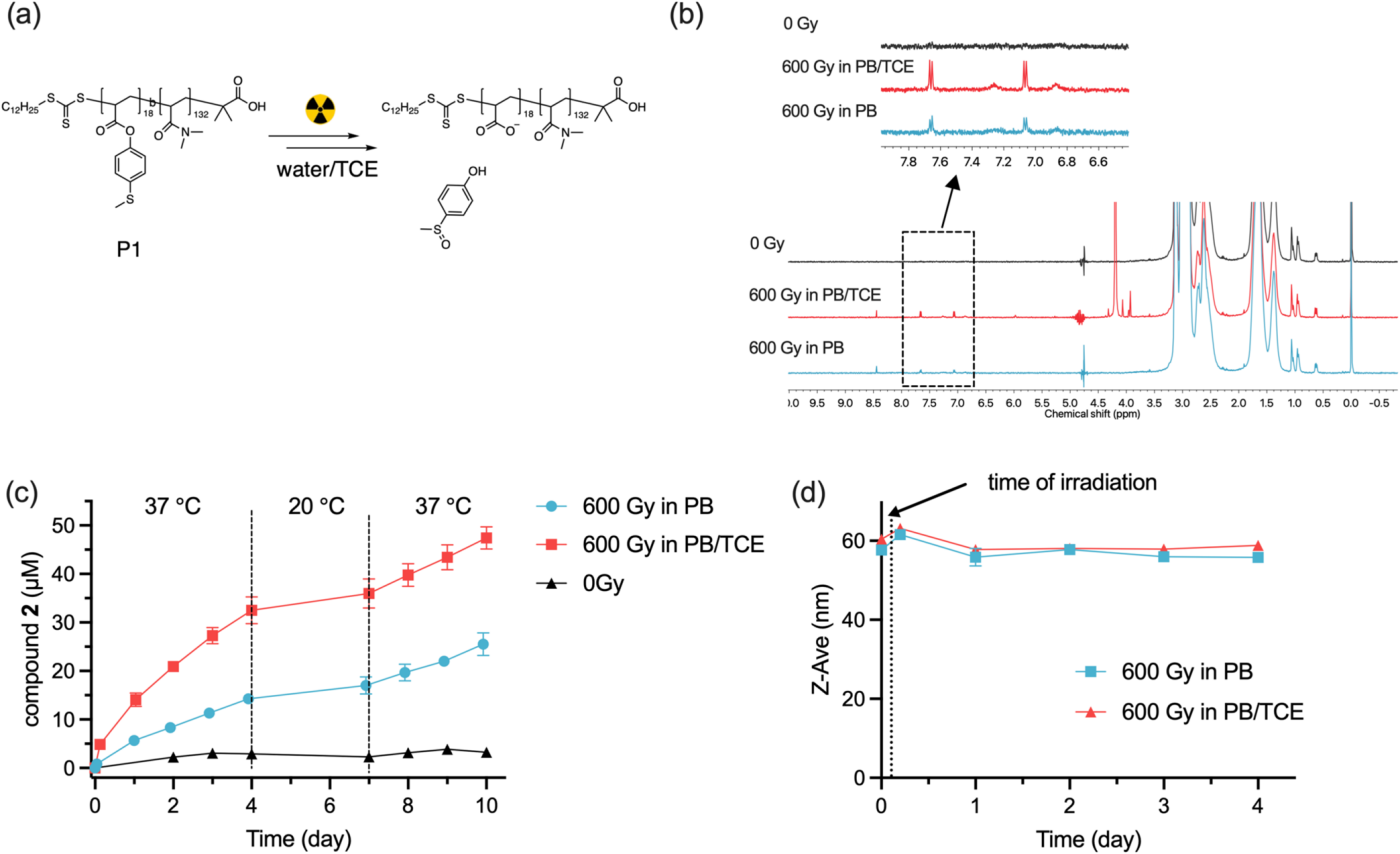
(a) Chemical structure of P1 and radiation-induced oxidation and hydrolysis. (b) ^1^H NMR of P1 aggregate solutions after exposure to 600 Gy of gamma-rays (spectra were taken after 37 °C incubation for 4 days). (c) Time evolution of compound **2** release after irradiation, quantified by the integration of the ^1^H NMR doublet at 7.06 ppm. (d) Time evolution of Z-average size of P1 aggregates after 600 Gy of γ-irradiation in PB and PB/0.1 vol % TCE, samples were incubated at 37 °C.

Having confirmed the oxidation of a small-molecule thioether, we then investigated whether the oxidation occurs when the thioether group is grafted to a polymer chain. We synthesized an amphiphilic block copolymer (P1, Figure 3a) which incorporates 4-(methylthio)phenyl acrylate (MTPA) as the hydrophobic monomer and *N*,*N*-dimethylacrylamide (DMA) as the hydrophilic monomer. The *M*_n_ is 9.9 kDa (*M*_w_ = 10.6 kDa, dispersity index *D̵* = 1.08, Figure S1) determined by GPC and the molecular weight is 16.9 kDa determined by ^1^H NMR. In PB (100 mM, pH 7.4), P1 self-assembles to form micelles (Figure S5b) with a hydrodynamic diameter (*D*_H_) of 57.7 ± 0.7 nm (Figure S3a, measured by DLS). After exposure to 600 Gy of γ-radiation in deuterated PB (*d*-PB, 100 mM, pD 7.4), micelle solutions (polymer concentration, 1.0 mg/mL) demonstrated a gradual release of compound **2**, as evidenced by the measured increase of peak intensity at 7.66 and 7.06 ppm (Figure 3b). Using sodium trimethylsilylpropanesulfonate (DSS) as an internal standard, we determined the concentration of released compound **2** (Figure 3c). Irradiation (600 Gy) in *d*-PB resulted in 0.8 ± 0.7 μM compound **2** released after 1 h of postirradiation incubation, while the release increased to 14.3 ± 0.7 μM after incubation at 37 °C for 4 days. The same dose of radiation in *d*-PB/TCE resulted in 4.9 ± 0.2 μM compound **2** released after 3 h of incubation and increased to 32.5 ± 2.8 μM after 4 days. The compound **2** release in *d*-PB is attributed to oxidation by hydrogen peroxide generated from water radiolysis. In the presence of 0.1 vol % of TCE, a stronger oxidizing species was generated, resulting in increased sulfoxide formation and release. The micelle solution was incubated at 20 °C from day 4 to day 7, which resulted in a lower rate of sulfoxide release. The *D*_H_ and the scattered counts were measured following radiation and incubation at 37 °C (Figures 3d and S4a). Although irradiation of aggregate in PB/TCE led to more compound **2** release compared to that in PB, the *D*_H_ and the scattered counts evolution of P1 aggregates after irradiation followed the same trend in both PB and PB/TCE. The *D*_H_ increased slightly after irradiation and returned to the original hydrodynamic diameter after 1 day of incubation, while the scattered counts dropped slightly after irradiation and remained unchanged following incubation. The cryogenic electron microscopy (cryo-EM) image of P1 after irradiation in PB/TCE demonstrated a similar microstructure compared to that before irradiation (Figure S5b,e). These results are attributed to the low ratio of thioether oxidation versus unreacted thioether. For instance, 2.0 mg/mL P1 contains approximately 2.2 mM thioether monomer. However, after exposure to 600 Gy of gamma-radiation in *d*-PB/TCE and 4 days of incubation, only 32.5 μM sulfoxide was released (Table 1). By this calculation, only 1.5% of the hydrophobic (methylthio)phenyl ester groups converted to hydrophilic carboxylic acid groups, which is likely insufficient to trigger micelle disassembly.

**Table 1:** Conversion from Thioether-Functionalized Polymers to Compound **2** after 600 Gy of Irradiation and 4 days Incubation

	[polymer] (mg/mL)	[thioether monomer] (mM)	solvent	[2] after 4 days (μM)	conversion (%)
P1	2.0	2.2	*d*-PB	14.3	0.65
			*d*-PB/TCE	32.5	1.5
P2	2.0	1.1	*d*-PB	5.60	0.51
			*d*-PB/TCE	18.4	1.7
P3	2.0	0.96	*d*-PB	6.18	0.64
			*d*-PB/TCE	18.2	1.9
rP2	2.0	0.92	*d*-PB	13.0	1.4

**Figure 4:**
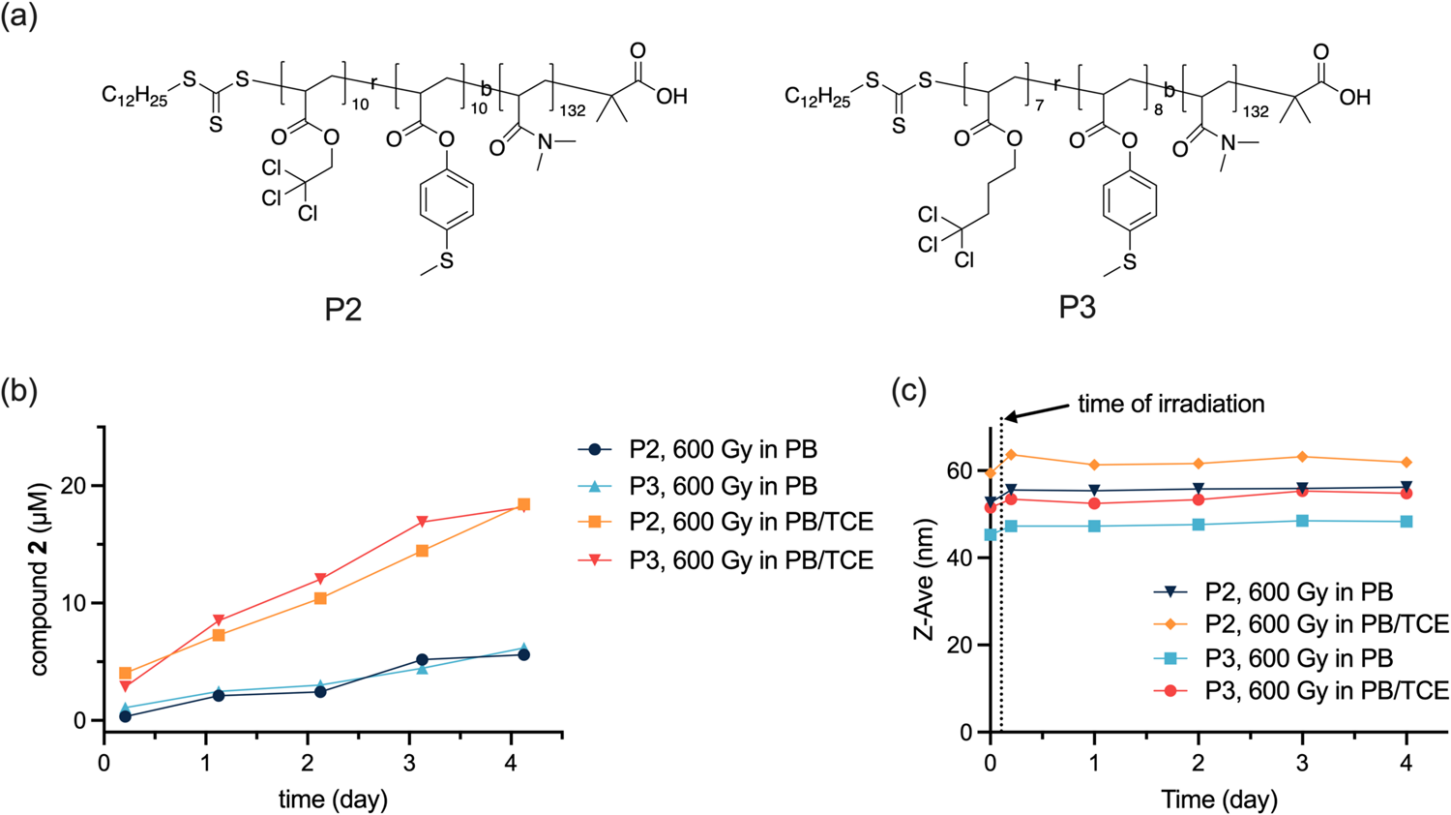
(a) Chemical structure of P2 and P3. (b) Time evolution of compound **2** release after radiation. (c) Time evolution of Z-average diameter of P2 and P3 after irradiation.

Although P1 micelles seem not to disassemble after exposure to radiation in *d*-PB/TCE, we wanted to investigate the behavior of these micelles when the alkyl chloride is covalently bound to the hydrophobic block. To investigate this, we synthesized block copolymers P2 and P3, where the alkyl chloride and MTPA are randomly copolymerized in the hydrophobic block (Figure 4a). P2 incorporates 2,2,2-trichloroethyl acrylate as the alkyl chloride, while P3 incorporates 4,4,4-trichlorobutyl acrylate. In PB, P2 and P3 self-assemble into micelles with *D*_H_ values of 52.6 ± 0.6 and 45.3 ± 0.2 nm, respectively (Figure S3c,e). Following exposure to 600 Gy of γ radiation in *d*-PB, P2 and P3 demonstrated similar sulfoxide release profiles, with approximately 5 μM compound **2** detected after 4 days of incubation at 37 °C, reaching a conversion of only ca. 0.6% (Table 1). In comparison, P1 had comparable conversion after the same dose of irradiation, meaning that the alkyl chloride attached to the hydrophobic block of the polymer has no enhancing effect on the oxidation of thioether groups. Addition of 0.1 vol % TCE to the P2 and P3 micelle solutions before irradiation significantly enhanced compound **2** release, with the conversion increasing to 2% (Figure 4b, Table 1). These results suggest that observed oxidation in the absence of TCE is related to H_2_O_2_ formation and that the covalent modification of the hydrophobic block with chlorinated side chains does not lead to increased oxidation. The *D*_H_ values of P2 and P3 aggregates increased slightly after irradiation and remained unchanged after 4 days of incubation irrespective of the presence of additional TCE (Figure 4c). P2 micelles were still visible in Cryo-EM after exposure to 600 Gy of γ-irradiation in PB/TCE (Figure S5f), indicating that no micelle disassembly occurred following radiation exposure.

**Figure 5:**
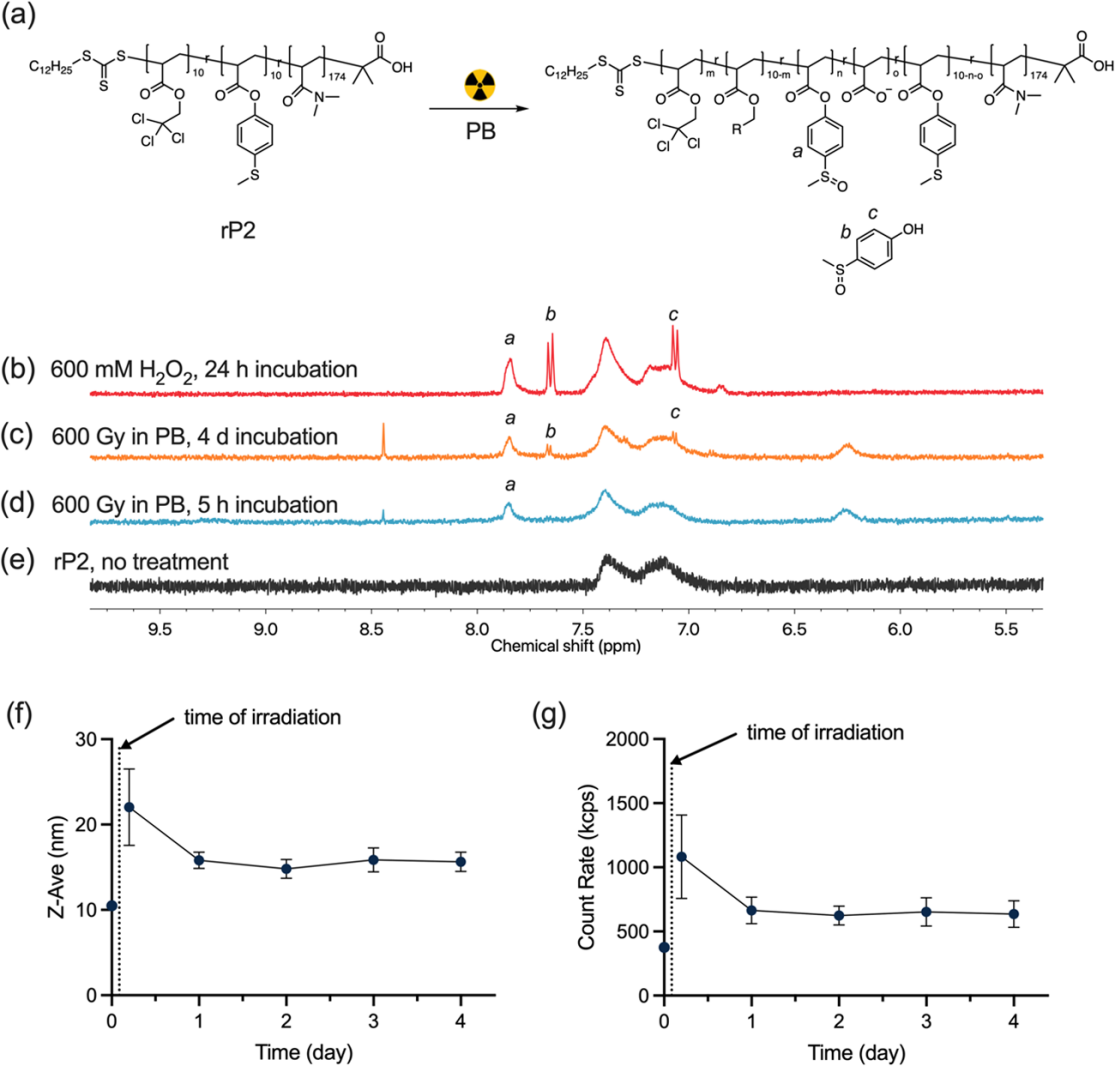
(a) Reaction scheme of rP2 solution when exposed to gamma-radiation, *R* indicates some fraction of converted chlorinated monomer. ^1^H NMR spectrum of rP2 in *d*-PB (100 mM, pD 7.4) (b) after adding 600 mM H_2_O_2_, (c) after exposure to 600 Gy gamma-radiation and incubation at 37 °C for 4 days, (d) after exposure to 600 Gy gamma-radiation and incubation at 37 °C for 5 h, (e) without further treatment. Time evolution of (f) Z-average diameter and (g) scatter count of rP2 after 600 Gy of γ-irradiation.

Apparently, alkyl chloride groups located in the hydrophobic core of the aggregates cannot enhance the radiation-induced thioether oxidation. We hypothesize that the penetration of aqueous electrons into the hydrophobic core of the aggregates is inhibited, and these electrons will be scavenged by dissolved molecular oxygen before reacting with the alkyl chloride groups. To investigate this hypothesis, we synthesized a copolymer (rP2) where DMA, MTPA, and 2,2,2-trichloroethyl acrylate are randomly copolymerized (Figure 5a). Because of the absence of blocks and the high ratio of the hydrophilic DMA repeating unit, rP2 has good solubility (more than 2 mg/mL) in PB (100 mM, pH 7.4). The appearance of peaks of the polymer aromatic groups in the ^1^H NMR spectrum (7.40–7.10 ppm, Figure 5e) indicates that the hydrophobic units are solvated. As shown in Figure 5b, a broad peak “*a*” (7.84 ppm) and two sharp doublet peaks “*b*, *c*” (7.65 and 7.06 ppm, respectively) were observed after the addition of 600 mM hydrogen peroxide. Peak “*a*” can be assigned to the poly(4-(methylsulfinyl)phenyl acrylate) while peak “*b*” and “*c*” can be assigned to compound **2**. However, peak “*a*” was not observed after adding 42 μM H_2_O_2_ (the expected amount of H_2_O_2_ produced by 600 Gy irradiation) and the same time incubation (Figure S6a), indicating a slow oxidation of thioether groups by H_2_O_2_ at low concentration. After exposure of rP2 (2 mg/mL in *d*-PB) to 600 Gy gamma-radiation following incubation at 37 °C for 5 h, peak “*a*” was observed while peak “*b*” and “*c*” were absent. This suggests a high rate of oxidation by the peroxyl radical generated from the 2,2,2-trichloroethyl ester and a relatively lower rate of ester hydrolysis. Notably, peak “a” was also observed when *t*-butyl alcohol (hydroxyl radical scavenger) was present during irradiation (Figure S7), meaning that hydroxyl radicals do not contribute significantly to thioether oxidation. After incubation for 4 days, 4-(methylsulfinyl)phenyl ester was slowly hydrolyzed to release compound **2** (Figure 5c). The concentration of the 4-(methylsulfinyl)phenyl ester repeating unit was 115 μM after gamma-irradiation (600 Gy) and 5 h of incubation (Figure 5d). The lower radiolytic yield of the 4-(methylsulfinyl)phenyl ester (0.19 μM/Gy) than the reported yield of aqueous electrons (0.28 μM/Gy) is likely caused by side reactions of aqueous electrons with dissolved molecular oxygen or other scavengers. After 4 days of incubation, the compound **2** concentration was 13 μM, which is more than two times higher than that generated from P2 and P3 under the same conditions. The oxidation was also observed after low dose irradiation (60 Gy) (Figure S6b). rP2 formed aggregates in PB with a *D*_H_ of 10.5 ± 0.1 nm (DLS, Figure S8). The *D*_H_ increased to 22.0 ± 4.5 nm after 600 Gy of γ-irradiation and equilibrated to 15.8 ± 1.0 nm after 1 day of incubation (Figure 5f). The count rate showed the same trend as that of *D*_H_ (Figure 5g). The observed initial response of micelles to radiation suggests that the structures have changed and it takes some time for them to equilibrate. Cryo-EM showed similar results, with spherical structures of 24.2 ± 3.6 nm that suggest micelle formation. The size of these particles increased slightly to 30.6 ± 5.8 nm after exposure to 600 Gy radiation. The scattered light counts increased from (3.8 ± 0.1) × 10^5^ to (10.8 ± 3.2) × 10^5^ cps (counts per second) after irradiation and regulated to (6.6 ± 1.0)×10^5^ cps after 1 day of incubation. The oxidation of rP2 and the high yield of compound **2** prove that the alkyl chloride groups that are covalently bound to the polymer chains are able to enhance the oxidation of the thioether.

## 4 Conclusions

We show that the reactive oxygen species generated from irradiated aqueous solutions containing alkyl chloride can oxidize thioether compounds to form sulfoxides. The oxidation also works if the thioether is covalently bound to an amphiphilic block copolymer. In aqueous solutions containing a small-molecule alkyl chloride, the peroxyl radical formed in the bulk solvent can diffuse into the hydrophobic core and oxidize the core component. However, the oxidation yield is severely reduced if the alkyl chloride is covalently bound to the hydrophobic block on the inside of polymer aggregates, since aqueous electrons formed in bulk solution are too hydrophilic to enter into the core of aggregates and instead will be scavenged by oxygen. Nevertheless, provided that the alkyl chloride groups bound to a polymer are not shielded from solution, they can react with aqueous electrons and form peroxyl radicals that can oxidize thioether groups. The findings presented in this work provide a rational design strategy for radiation-sensitive polymer materials that can be used in radiation-induced oxidation and drug release systems.

## Supporting Information Available

Experimental details and materials; synthesis procedures; ^1^H and ^13^C NMR spectrum for all compounds; and GPC chromatographs, cryo-EM images, and DLS size distribution measurements (PDF)
