# Supplementary material for: Alkyl Chloride-Functionalized Polymers Mediate Oxidation of Thioethers Initiated by Ionizing Radiation
Source: ACS Appl Polym Mater. 2025 Mar 19;7(6):3835–41. doi: 10.1021/acsapm.5c00054 (PMC11959515; doi:10.1021/acsapm.5c00054)
Supplement: Supplementary file 1 — ap5c00054_si_001.pdf [file ap5c00054_si_001.pdf]

Supporting Information

Alkylchloride-functionalized polymers mediate oxidation of thioethers initiated by ionizing radiation

Juncheng Liu[1,2], Irene Piergentili[1], Bing Xu[1,2], Antonia G. Denkova[2,*] and Rienk Eelkema[1,*]

[1] Department of Chemical Engineering, Delft University of Technology, van der Maasweg 9, 2629 HZ Delft

[2] Department of Radiation Science and Technology, Delft University of Technology, Mekelweg 15, 2629 JB Delft

*Corresponding Authors: Antonia G. Denkova and Rienk Eelkema, Email: A.G.Denkova@tudelft.nl; R.Eelkema@tudelft.nl

Synthesis

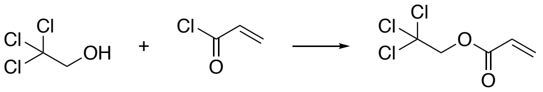

S1

Acryloyl chloride (2.2 g, 1 eq.), 1,1,1-trichloroethanol (4.1 g, 1.1 eq.) and DCM (30 mL). were added to a flame dried Schlenk flask. The mixture was degassed with N_2 and cooled to -20 °C using an ethanol/liquid nitrogen bath. Triethylamine (3.05 g, 1.2 eq) was added slowly. The mixture was allowed to return to room temperature and stirred overnight. The reaction was quenched by adding 1 M HCl (100 mL). The organic phase was washed with brine 3 times and dried with $MgSO_4$. The solvent was evaporated under reduced pressure. The crude product was purified using flash column chromatography eluted with PE/DCM = 10/1. The product was collected as a colorless oil (yield 4.85 g, 86.9%). ^{1}H-NMR (399.67 MHz, $CDCl_3$, ppm) δ = 6.56 (dd, J_1 = 1.16 Hz, J_2 = 17.32 Hz, 1H), 6.22 (dd, J_1 = 17.32 Hz, J_2 = 10.48 Hz, 1H), 5.99 (dd, J_1 = 1.12 Hz, J_2 = 10.48 Hz, 1H), 4.81 (s, 2H). ^{13}C NMR (99.91 MHz, $CDCl_3$, ppm) δ = 164.30, 133.00, 127.03, 94.88, 74.03. Spectroscopic data corresponds to data reported in literature. [1]

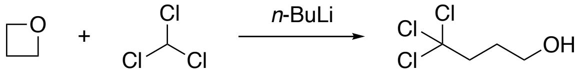

S2

Compound S2 was synthesized using the method reported by Tsuji et al. [2] Anhydrous chloroform (1.32 mL, 1.2 eq.) and THF (20 mL) were added to a flame dried Schlenk flask. The mixture was degassed with nitrogen and cooled to -95 °C using an ethanol/liquid nitrogen bath. *n*-butyllithium (1.6 M in hexane, 10.3 mL, 1.2 eq) was then slowly added, followed by adding $BF_3 \cdot OEt_2$ (2.04 mL, 1.2 eq.) and oxetane (895 µL, 1 eq.). The mixture was returned to room temperature and stirred overnight. The reaction was quenched by 1 M HCl and washed with brine 3 times. The organic phase was collected, dried over $MgSO_4$ and purified using flash column chromatography eluted with PE/EA = 5/1, yielding a yellow oil (673 mg, 27.5%). ^{1}H-NMR (399.67 MHz, $CDCl_3$, ppm) δ = 3.75 (t, J = 6.24 Hz, 2H), 2.81 (t, J = 8.12 Hz, 2H), 2.06-1.99 (m, 2H). ^{13}C NMR (99.91 MHz, $CDCl_3$, ppm) δ = 99.90, 61.18, 51.94, 29.61. Spectroscopic data corresponds to data reported in literature. [3]

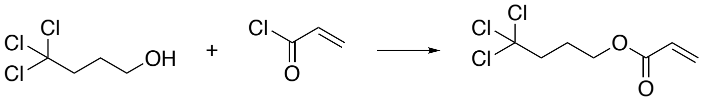

S3

Compound S3 was synthesized using the same method as compound S1, and collected as a colorless oil, yield 780 mg. ^{1}H-NMR (399.67 MHz, $CDCl_3$, ppm) δ = 6.41 (d, J = 17.36 Hz, 1H), 6.12 (dd, J_1 = 17.36 Hz, J_2 = 10.40 Hz, 1H), 5.84 (d, J = 10.40 Hz, 1H), 4.25 (t, J = 6.28 Hz 2H), 2.78 (t, J = 7.84 Hz, 2H), 2.19-2.12 (m, 2H). ^{13}C NMR (99.91 MHz, $CDCl_3$, ppm) δ = 165.95, 131.15, 128.08, 99.28, 62.67, 51.90, 25.99.

Synthesis of macroDDMAT

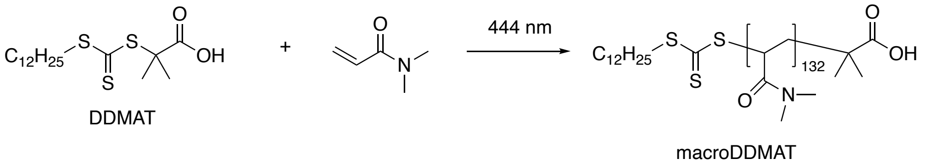

DDMAT (0.12 g, 1 eq.), basic alumina-filtered N,N-dimethylacrylamide (DMA, 6.90 g, 200 eq.) and 6.8 mL DMF were added in a 10 mL Schlenk flask. The solution was bubbled with nitrogen for 15 minutes and then sealed. ^{1}H NMR at t_0 was recorded. The reaction mixture was stirred for 6 hours at room temperature in a LED light reactor using 444 nm blue light. ^{1}H NMR was taken to determine the conversion of DMA. The reaction mixture was diluted with DCM (50 mL) and precipitated three times in diethyl ether (500 mL), after which the product was dried in a vacuum oven at 40 °C for 3 days to obtain 7.32 g of a light yellow powder. The degree of polymerization was calculated to be on average 132 DMA repeating units.

Synthesis of P1

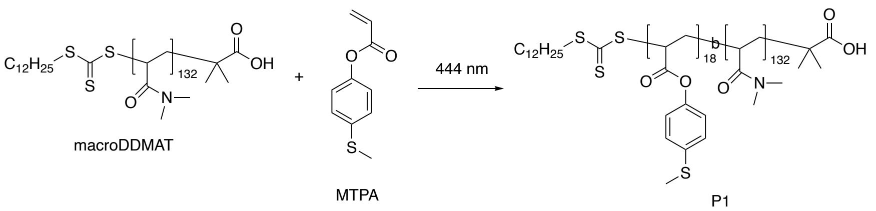

P1 was synthesized following an reported approach.[4] $M_{n,NMR}$ = 16.9 kDa, $M_{n,GPC}$ = 9.9 kDa, $M_{w,GPC}$ = 10.6 kDa, dispersity index $Đ$ = 1.08. .

Synthesis of P2

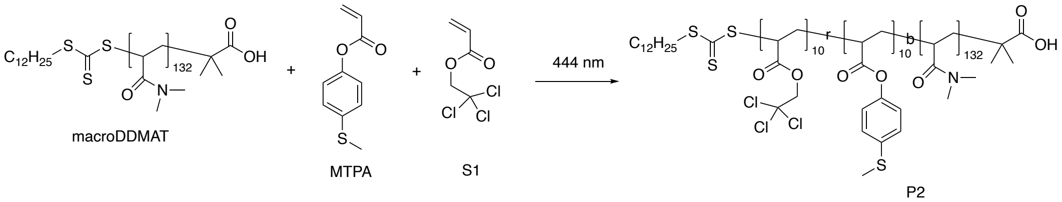

The used amount of chemicals in this synthesis is listed in **Table S1**. MacroDDMAT, MTPA and compound S1 were dissolved in DMF, followed by bubbling with nitrogen for 15 minutes. ^{1}H NMR at t_0 was recorded. The resulting solution was stirred in a light reactor (444 nm) for the given time. ^{1}H NMR was taken to determine the conversion of MTPA and

compound S1. After reaching the desired monomer conversion, the reaction mixture was diluted with DCM (50 mL) and precipitated three times in diethyl ether (500 mL). The product was filtrated and dried in a vacuum oven for 3 days (40 °C). P2 was collected as light-yellow powder (0.78 g). The degree of polymerization for MTPA and S1 are on average 10 repeating units. $M_{n,NMR}$ = 17.4 kDa, $M_{n,GPC}$ = 10.7 kDa, $M_{w,GPC}$ = 11.6 kDa, dispersity index $Đ$ = 1.08.

Synthesis of P3

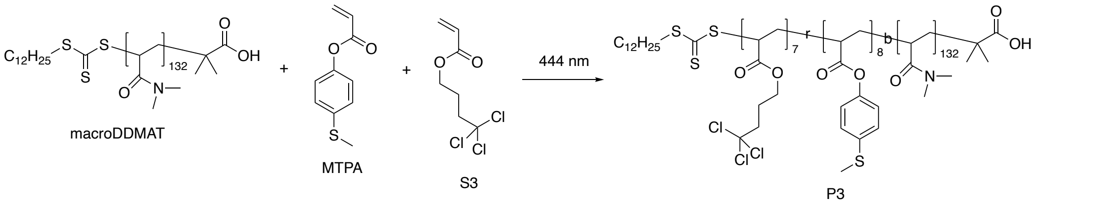

P3 was synthesized using the same method as P2. P3 was collected as light-yellow powder, yield 1.10 g. The degree of polymerization for MTPA and S3 are 8 and 7 repeating units, respectively. $M_{n,NMR}$ = 16.7 kDa, $M_{n,GPC}$ = 9.9 kDa, $M_{w,GPC}$ = 10.6 kDa, dispersity index $Đ$ = 1.07.

Synthesis of rP2

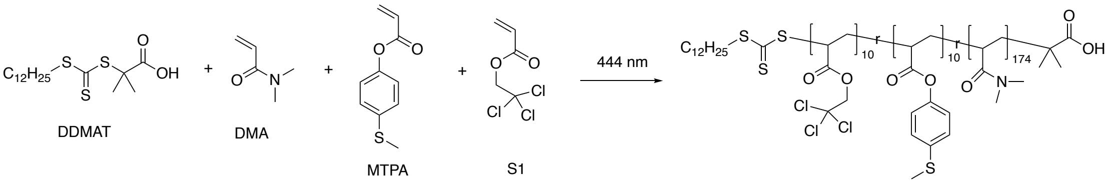

Specific amounts of DDMAT, DMA, MTPA, compound S1 and DMF were added in a 10 mL Schlenk flask. The solution was bubbled with nitrogen for 15 minutes and sealed. ^{1}H NMR at t_0 was recorded. The reacting mixture was stirred for 16 hours at room temperature in a LED light reactor giving blue light of 444 nm. ^{1}H NMR was taken to determine the conversion of each monomer. The reacting mixture was diluted with DCM (50 mL) and precipitated in diethyl ether (500 mL) three times, after which the product was dried in a vacuum oven at 40 °C for 3 days to obtain 1.73 g light yellow powder. The degree of polymerization was calculated to be 174 DMA units, 10 S1 units and 10 MTPA units. $M_{n,NMR}$ = 21.6 kDa, $M_{n,GPC}$ = 11.5 kDa, $M_{w,GPC}$ = 12.6 kDa, dispersity index $Đ$ = 1.09.

Table S1. P2, P3 and rP2 synthesis data.

	macro-DDMAT (g)	DDMAT (mg)	DMA (g)	MTPA (mg)	S1 (mg)	S3 (mg)	DMF (mL)	Reacting time (h)
P2	1.3	0	0	381	399	0	5	21
P3	1.3	0	0	381	0	498	5	11
rP2	0	34.8	2	197	206	0	2	16

Polymer characterization

For all polymers, molecular weight (M_n) and dispersity index ($Đ$) were characterized by gel

permeation chromatography (GPC) using the UV detector for analysis.

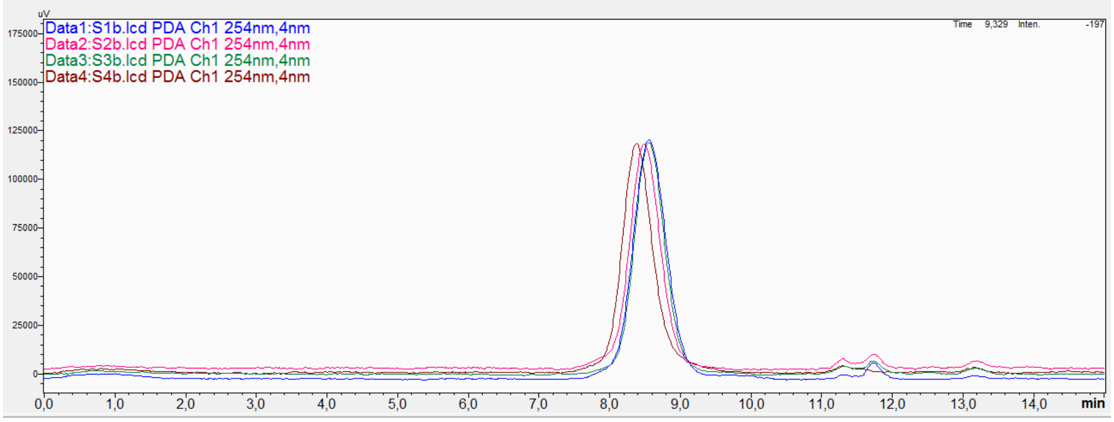

Figure S1. GPC chromatograms of P1 (data1), P2 (data2), P3 (data3) and rP2 (data4).

The polymer structure was derived from ^{1}H NMR studies as described below.
Polymerization conversion (*c*) was calculated by monitoring the integration of unsaturated protons ($\int M$: 5.66 ppm for DMA, 6.60 ppm for MTPA, 6.57 ppm for S1 and 6.43 ppm for S3) relative to that of DMF ($\int M$: 8.00 ppm). The conversion (*c*) of each monomer was calculated using the following equation:

$$c = \frac{\int M(t_0) - \int M(t)}{\int M(t_0)} \qquad S(1)$$

The degree of polymerization (*n*) of each monomer was calculated by:

$$n = \frac{M(monomer)_0}{M(CTA)_0} \times c \qquad S(2)$$

where $M(monomer)_0$ and $M(CTA)_0$ are the concentration of monomer and chain transfer agent at time zero of polymerization, respectively.
The molecular weight of a polymer with z monomers, $M_{n,conv}$ was calculated by:

$$M_{n,conv} = \sum_{x=1}^{z} c \times n \times M_x + M_{CTA} \qquad S(3)$$

where M_x and M_{CTA} are the molecular weight of the monomer and the chain transfer agent, respectively.

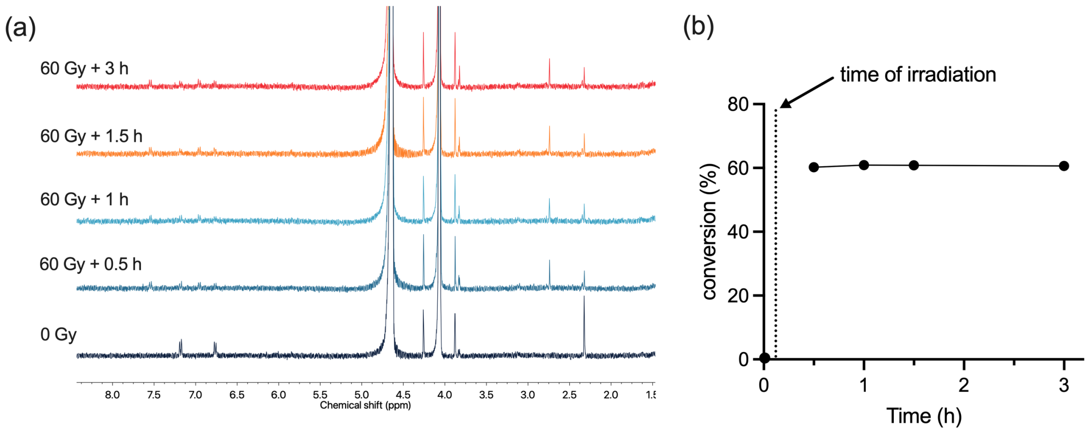

Figure S2. (a) ^{1}H NMR of compound **1** in D_2O/12 mM TCE after γ-irradiation; (b) the conversion of compound **2** versus incubation time.

Dynamic light scattering (DLS) measurements

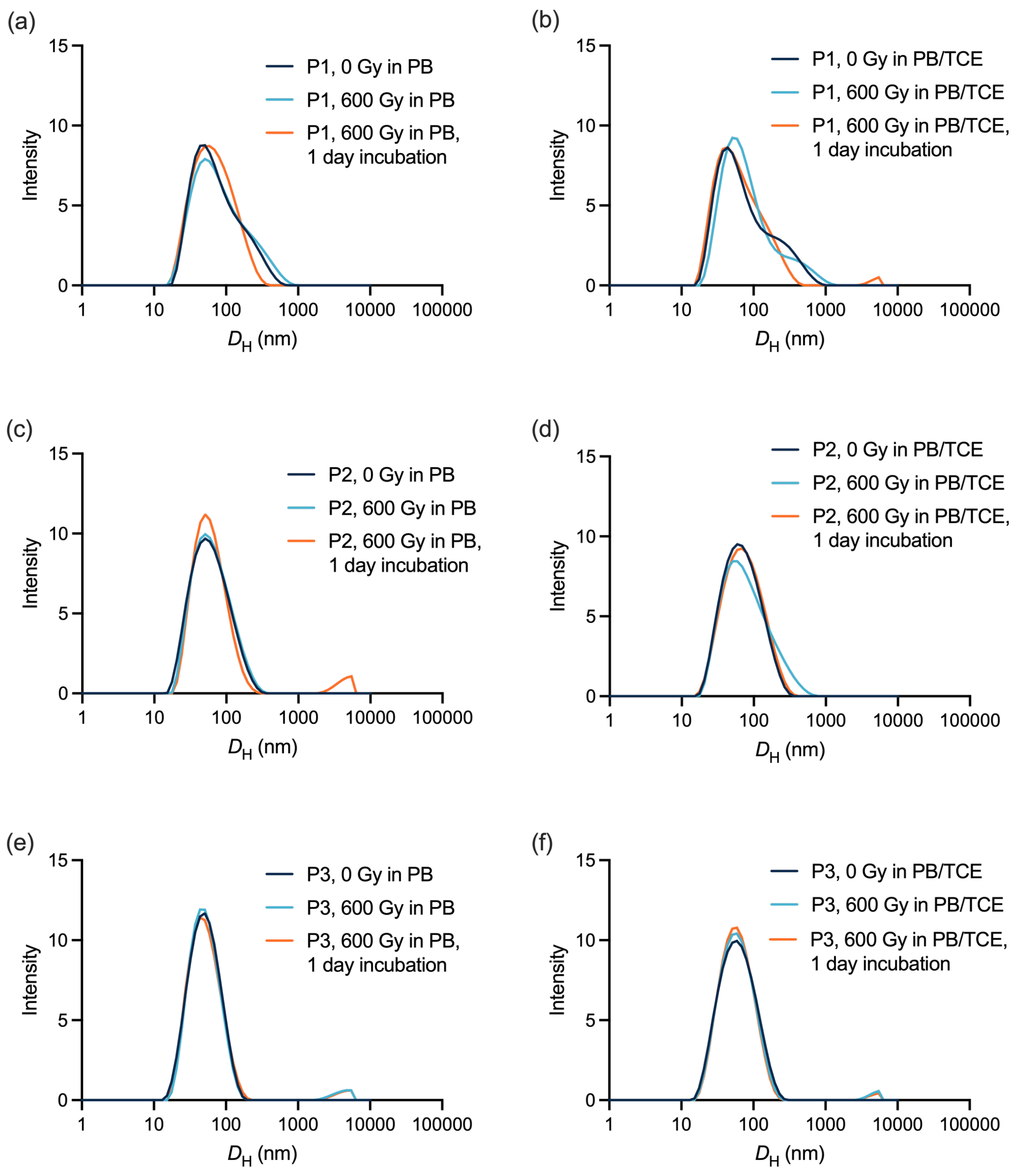

Figure S3. Intensity plot measured by DLS of polymer aggregates before irradiation, after 600 Gy γ-irradiation without incubation and after 600 Gy γ-irradiation with 37 °C incubation for 1 day. (a) P1 (1 mg/mL in PB, pH 7.4), (b) P1 (1 mg/mL in PB/0.1 vol% TCE, pH 7.4), (c) P2 (1 mg/mL in PB, pH 7.4), (d) P2 (1 mg/mL in PB/0.1 vol% TCE, pH 7.4), (e) P3 (1 mg/mL in PB, pH 7.4), (f) P3 (1 mg/mL in PB/0.1 vol% TCE, pH 7.4).

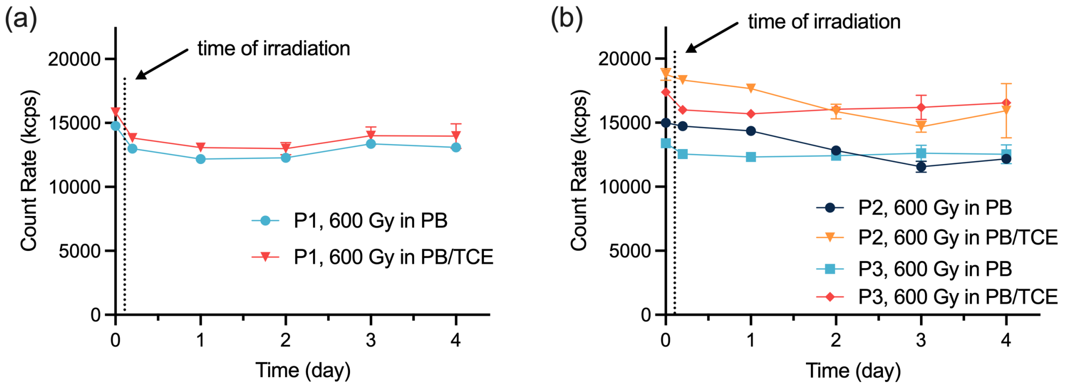

Figure S4. Time evolution of scatter count of polymer aggregates after 600 Gy γ-irradiation and 37 °C incubation. (a) P1 (1 mg/mL) in PB and PB/0.1 vol% TCE, (b) P2 (1 mg/mL) and P3 (1 mg/mL) in PB and PB/0.1 vol% TCE.

Micelle morphology determination using cryogenic electron microscopy (cryo-EM)
Polymer solutions of rP2, P1 and P2 were all prepared using the same method. 10 mg of polymer was dissolved in 0.1 mL THF followed by adding 1 mL PB under vigorous stirring. The solution was stirred for 24 h in an open vial to evaporate THF. After exposure to 600 Gy γ-radiation, solutions were incubated at 37 °C for 24 hours. Cryo-EM images were acquired using the following procedure: 3 μL of the solution was applied onto a Quantifoil 1.2/1.3 200 mesh Cu grid. The grid was blotted for 6-12 seconds with filter paper to create a thin layer, and then rapidly immersed in liquid ethane (Leica EM GP version 16222032) for vitrification. Subsequently, the grid was inserted into a cryo-holder (Gatan model 626) and transferred to the Jeol JEM 1400 TEM. Images were processed and analyzed using ImageJ.

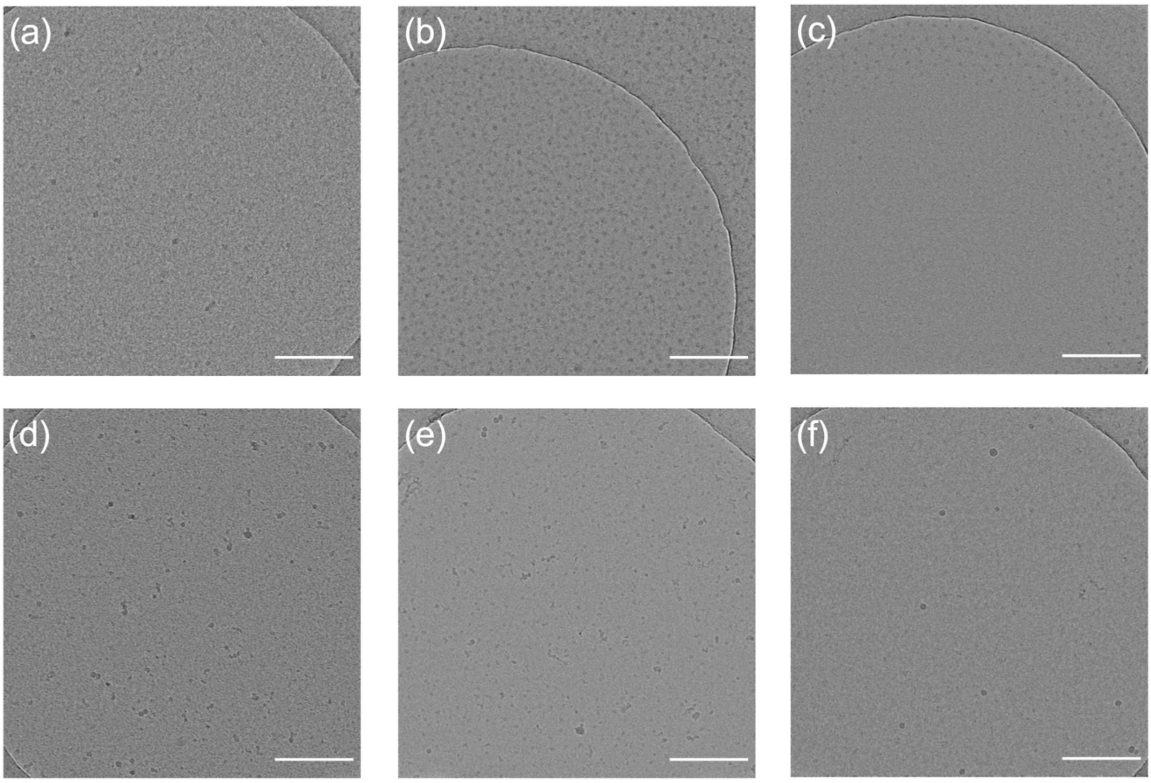

Figure S5. Cryo-EM images of (a) rP2, (b) P1 and (c) P2 before irradiation; (d) rP2, (e) P1 and (f) P2 after exposure to 600 Gy of γ-irradiation. Scale bar is 200 nm.

Table S1. Average micelle diameter measured from cryo-EM images (Figure S5).

Polymer	Diameter before irradiation (nm)	Diameter after 600Gy irradiation (nm)
rP2	24.2±3.6	30.6±5.8
P1	26.5±5.5	27.1±9.1
P2	21.1±4.7	27.2±6.0

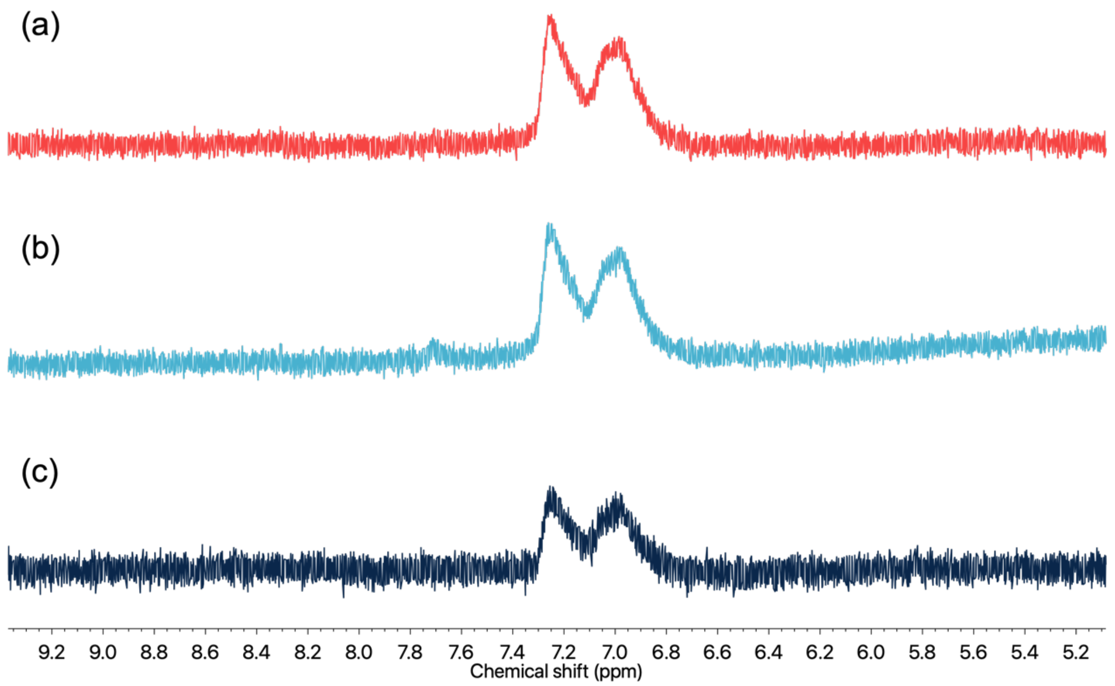

Figure S6. ^{1}H NMR spectrum of rP2 in D_2O (a) after addition of 42 μM of H_2O_2 and 37 °C incubation for 5 hours, (b) after exposure to 60 Gy γ-radiation and (c) without further treatment.

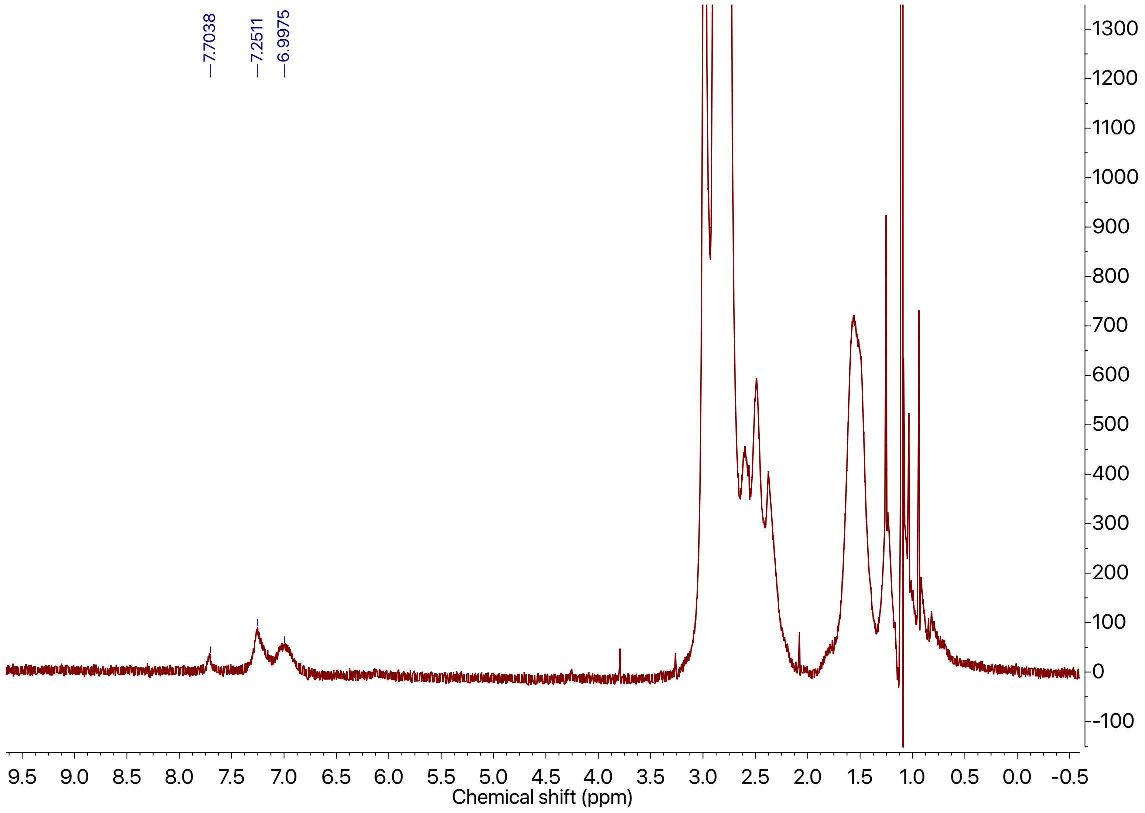

Figure S7. ^{1}H NMR spectrum of rP2 in *d*-PB/10 mM *t*-butyl alcohol after 600 Gy of

irradiation

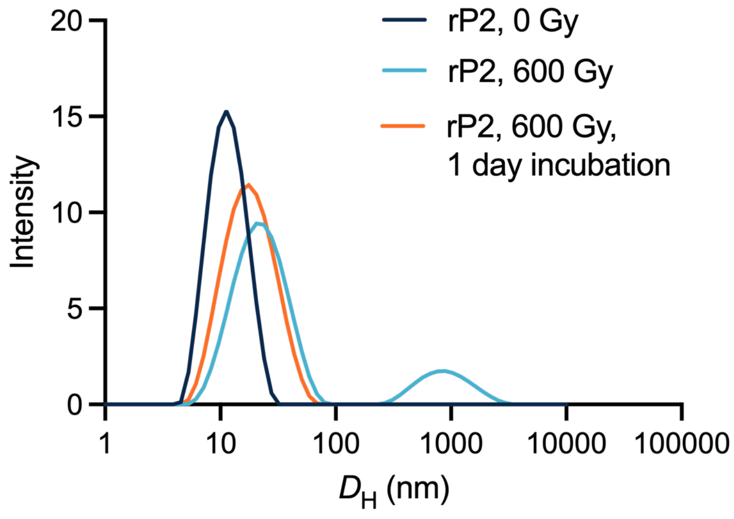

Figure S8. Intensity plot measured by DLS of rP2 before irradiation, after 600 Gy γ-irradiation without incubation and after 600 Gy γ-irradiation with 37 °C incubation for 1 day.

Degree of polymerization calculated by ^{1}H NMR spectroscopy.

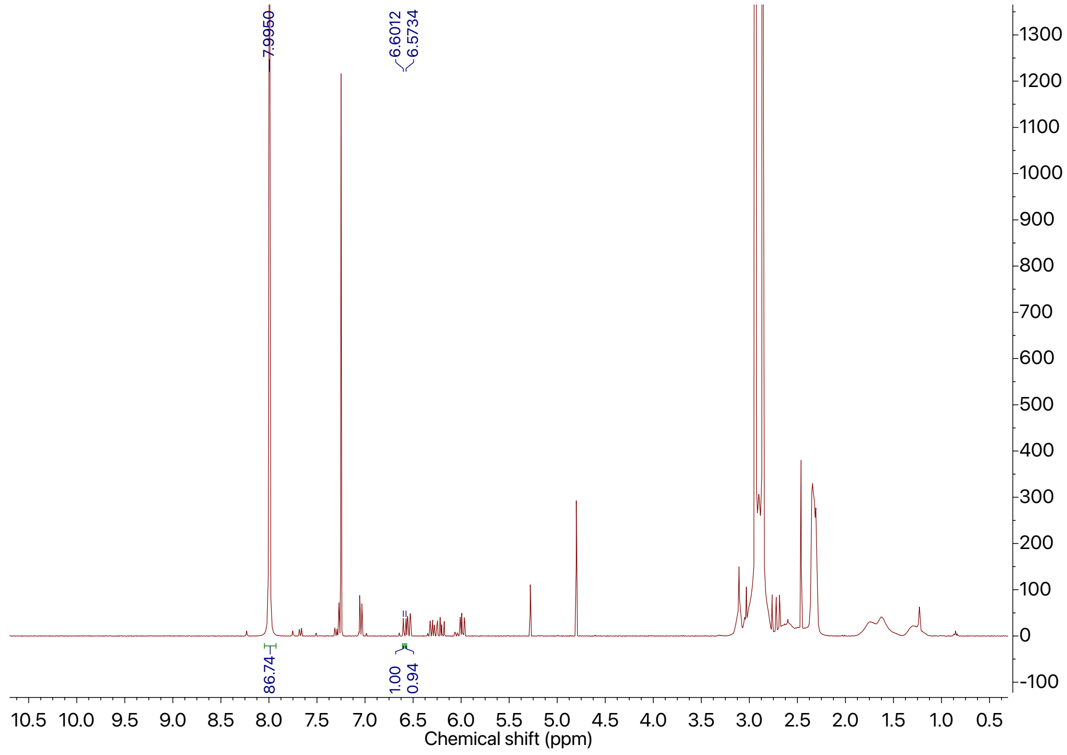

Figure S9. ^{1}H NMR of the polymerization mixture of P2 at t_0.

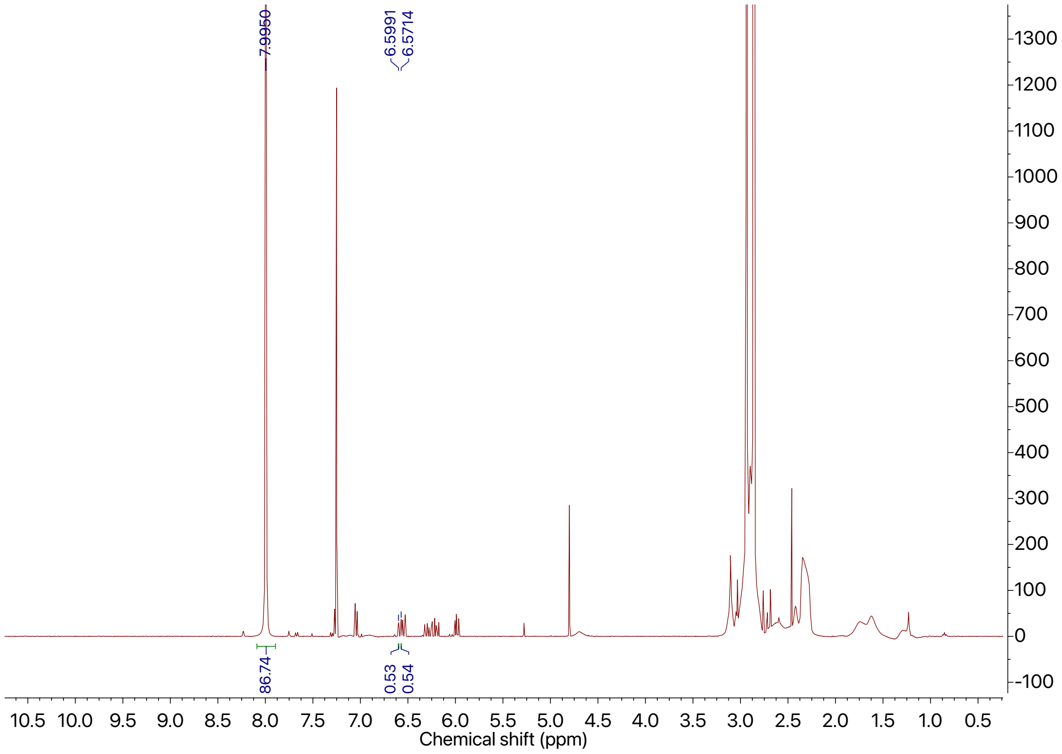

Figure S10. ^{1}H NMR of the polymerization mixture of P2 at t_{21h}.

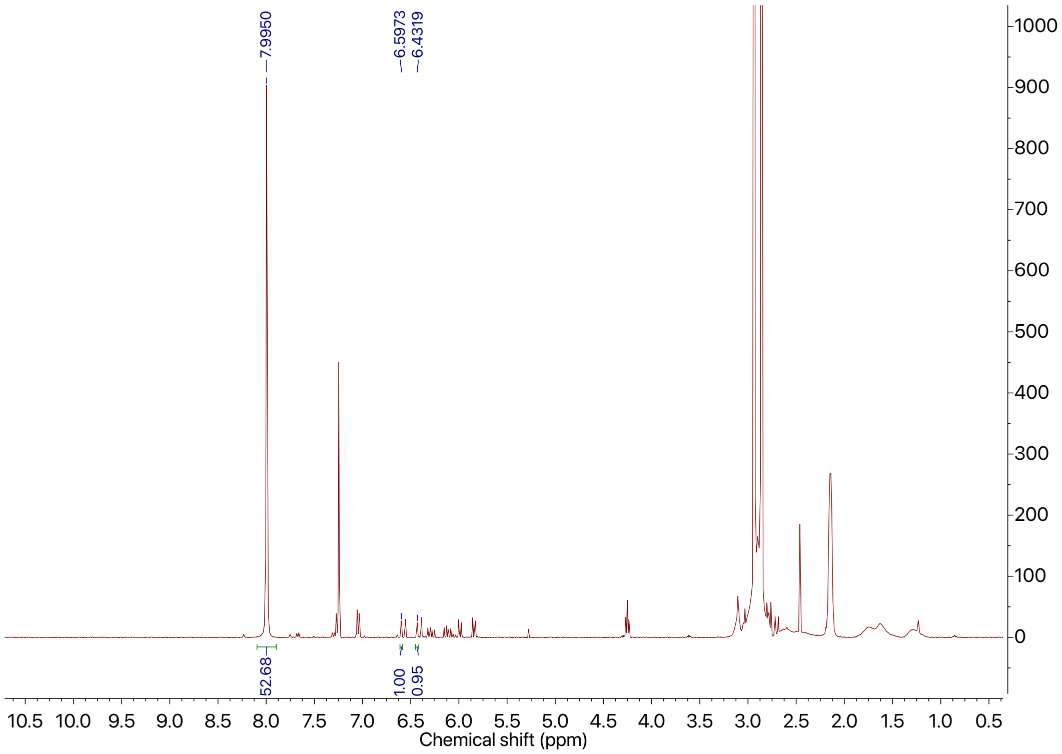

Figure S11. ^{1}H NMR of the polymerization mixture of P3 at t_0.

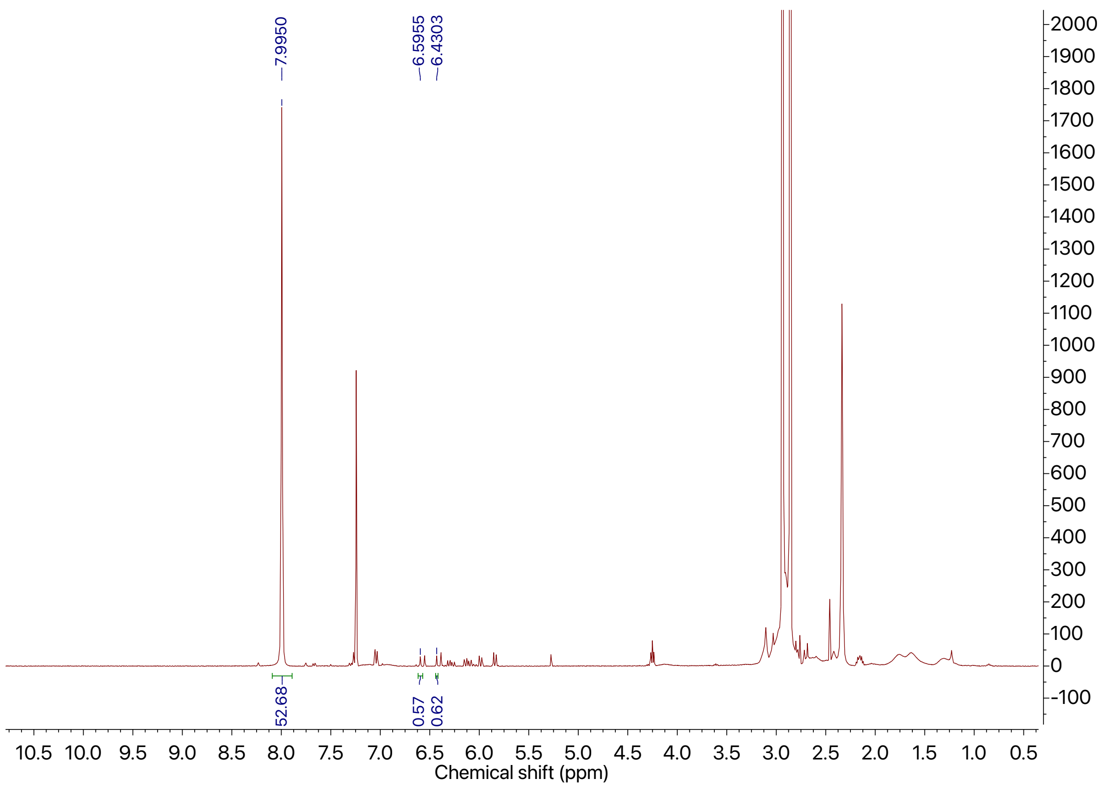

Figure S12. ^{1}H NMR of the polymerization mixture of P3 at t_{11h}.

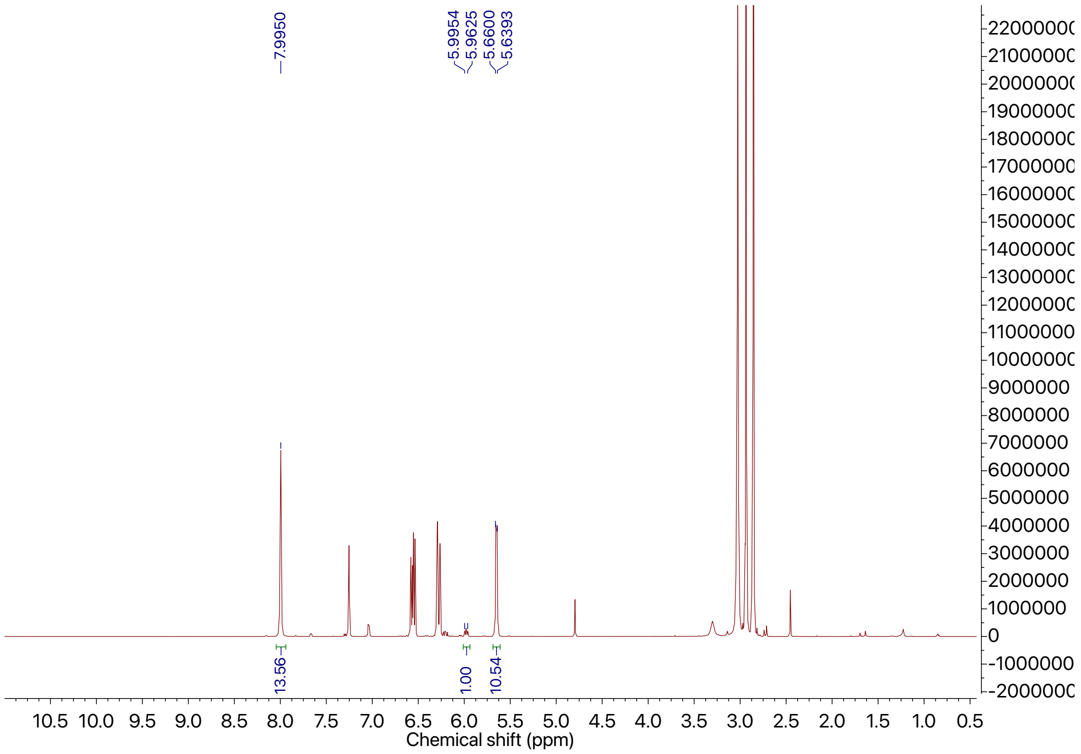

Figure S13. ^{1}H NMR of the polymerization mixture of rP2 at t_0.

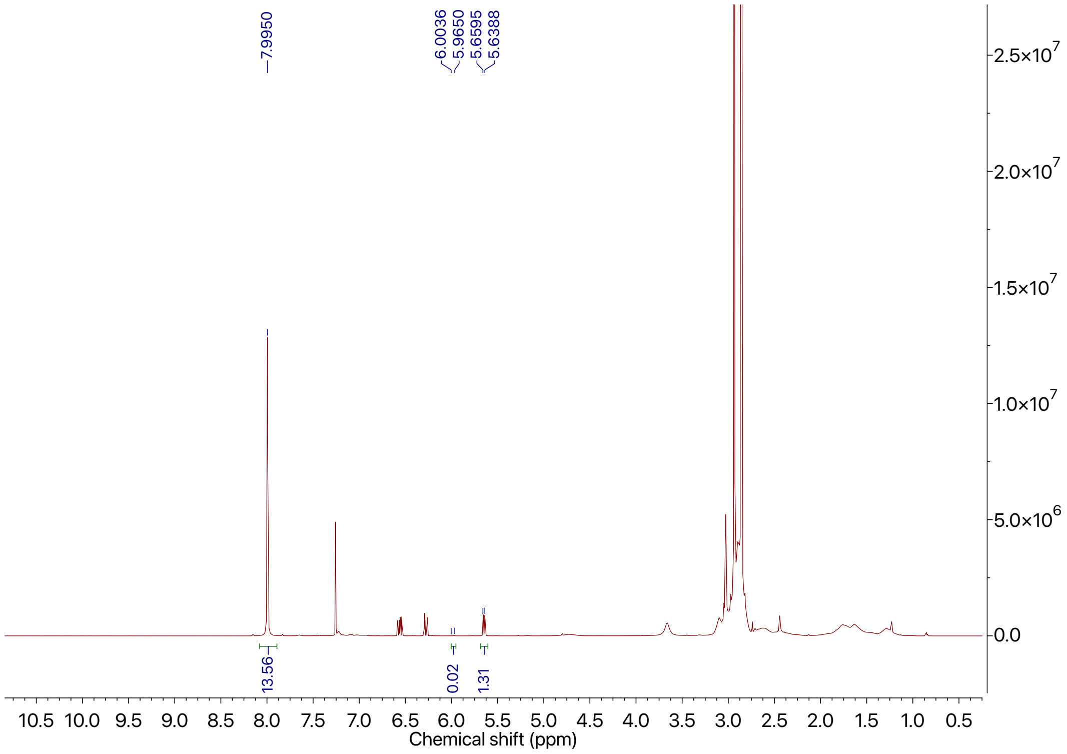

Figure S14. ^{1}H NMR of the polymerization mixture of rP2 at t_{16h}.

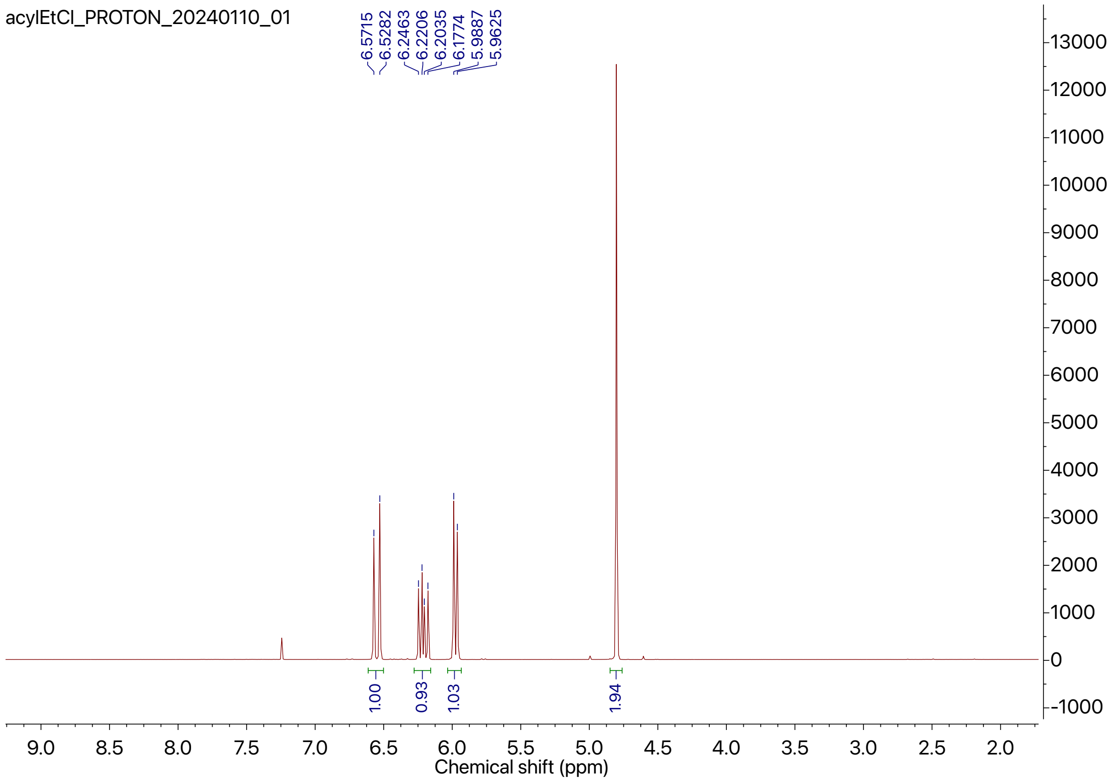

Figure S15. ^{1}H NMR spectrum of S1

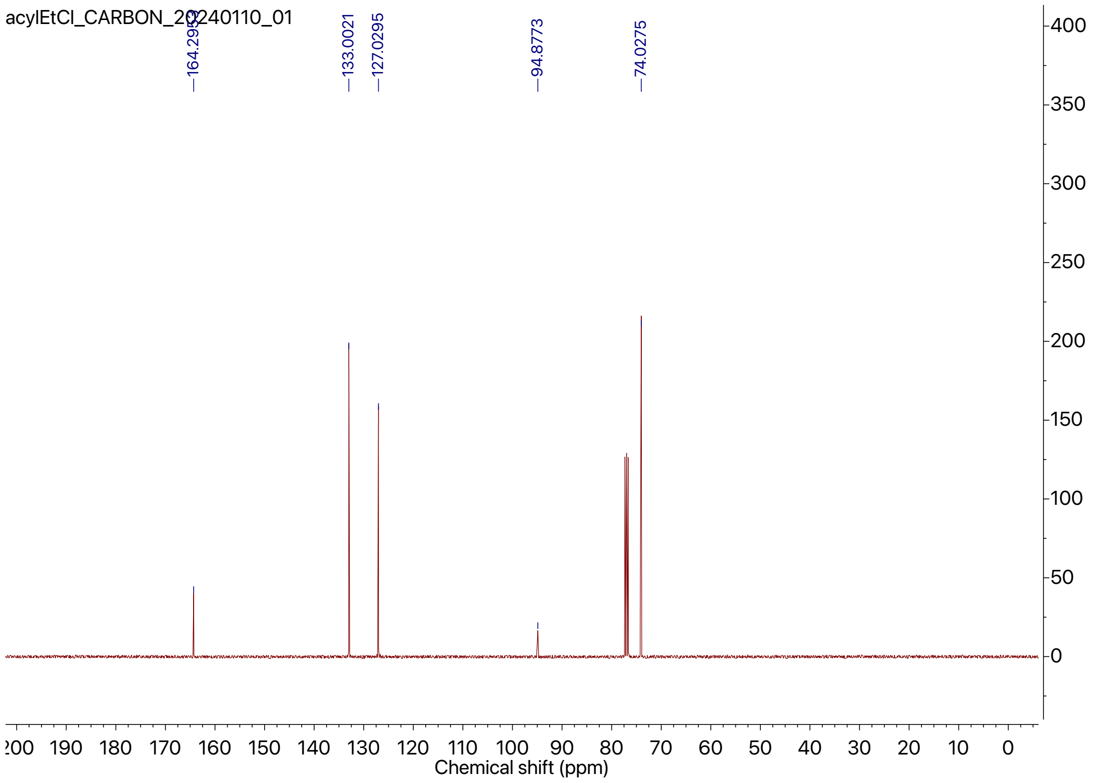

Figure S16. ^{13}C NMR spectrum of S1

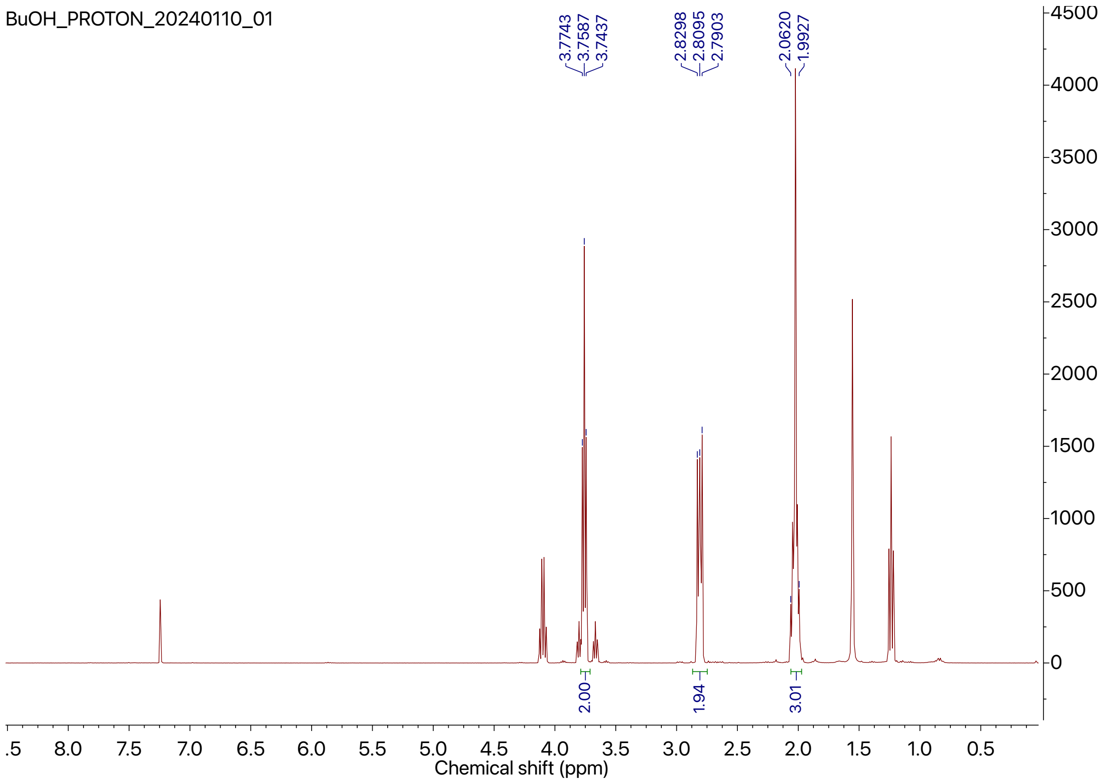

Figure S17. ^{1}H NMR spectrum of S2

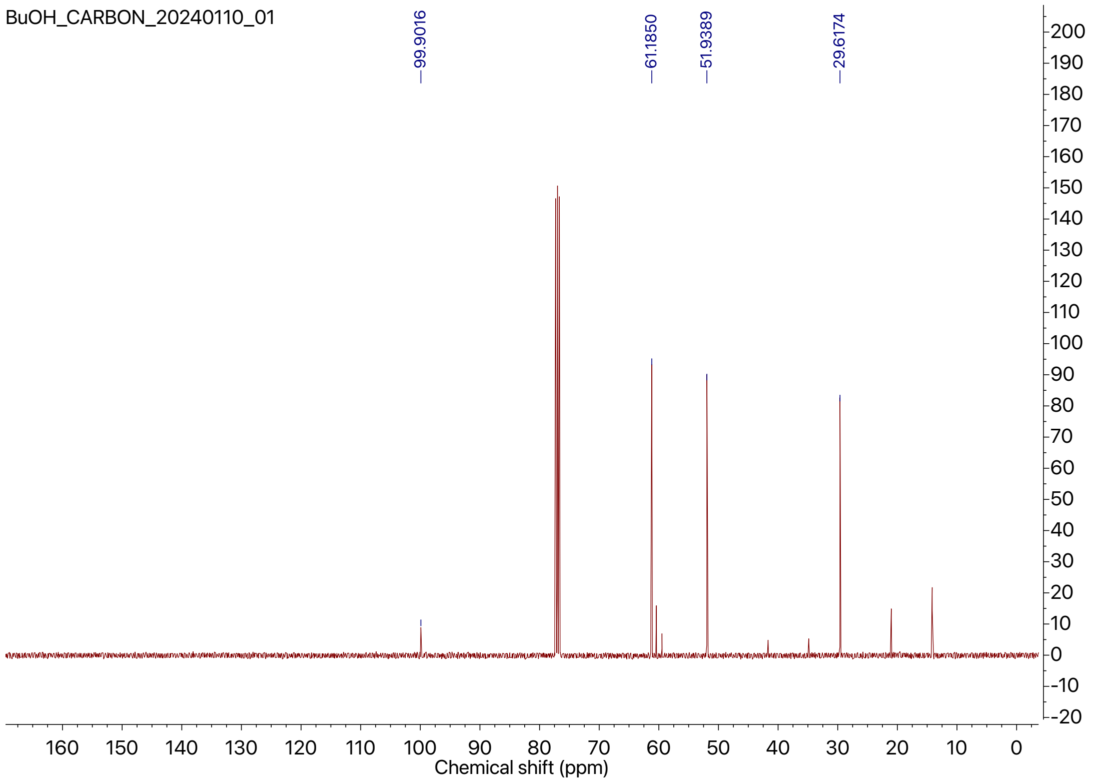

Figure S18. ^{13}C NMR spectrum of S2

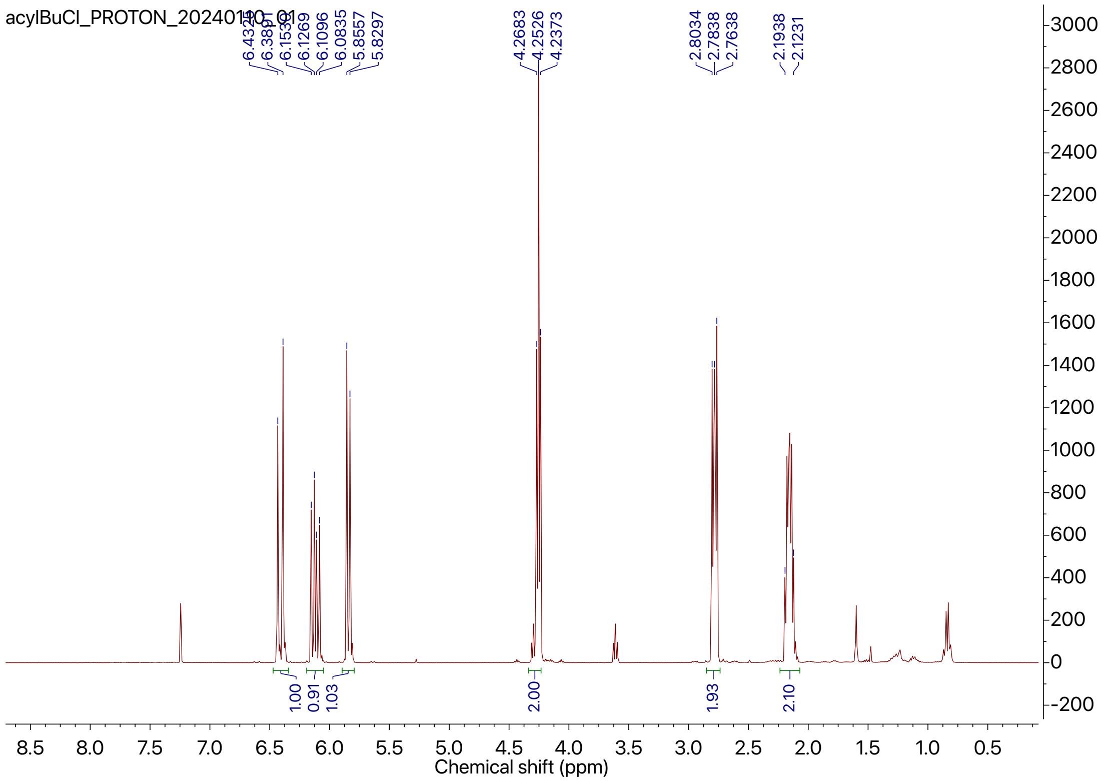

Figure S19. ^{1}H NMR spectrum of S3

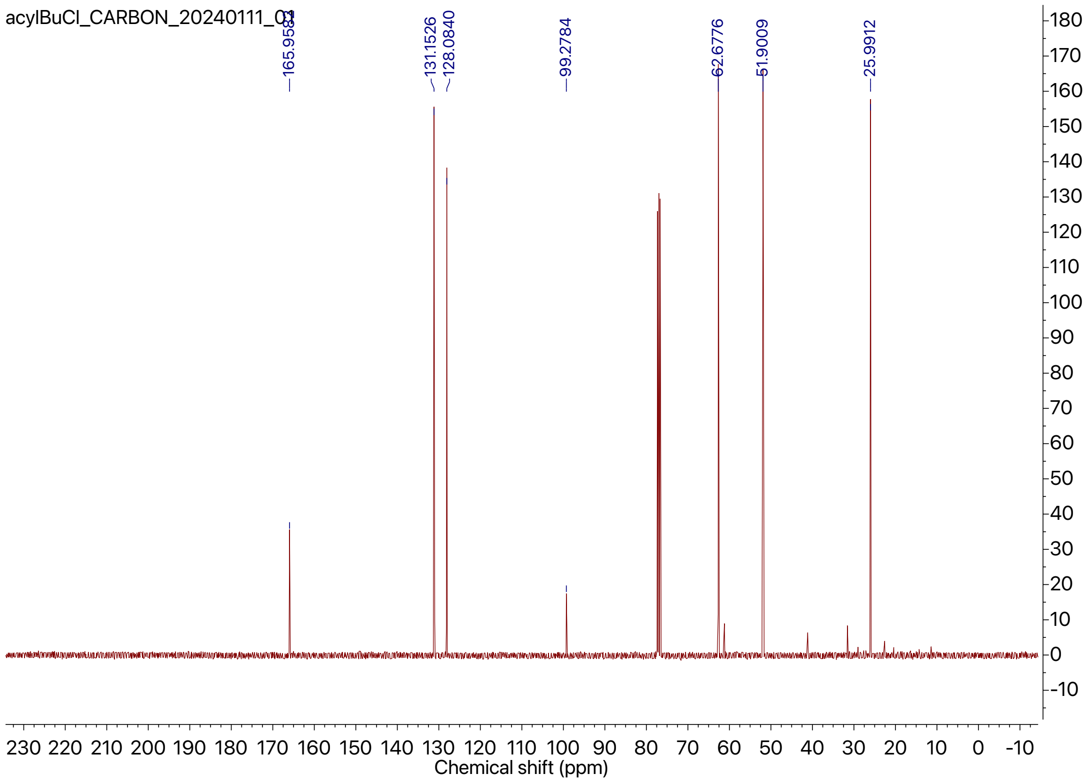

Figure S20. ^{13}C NMR spectrum of S3

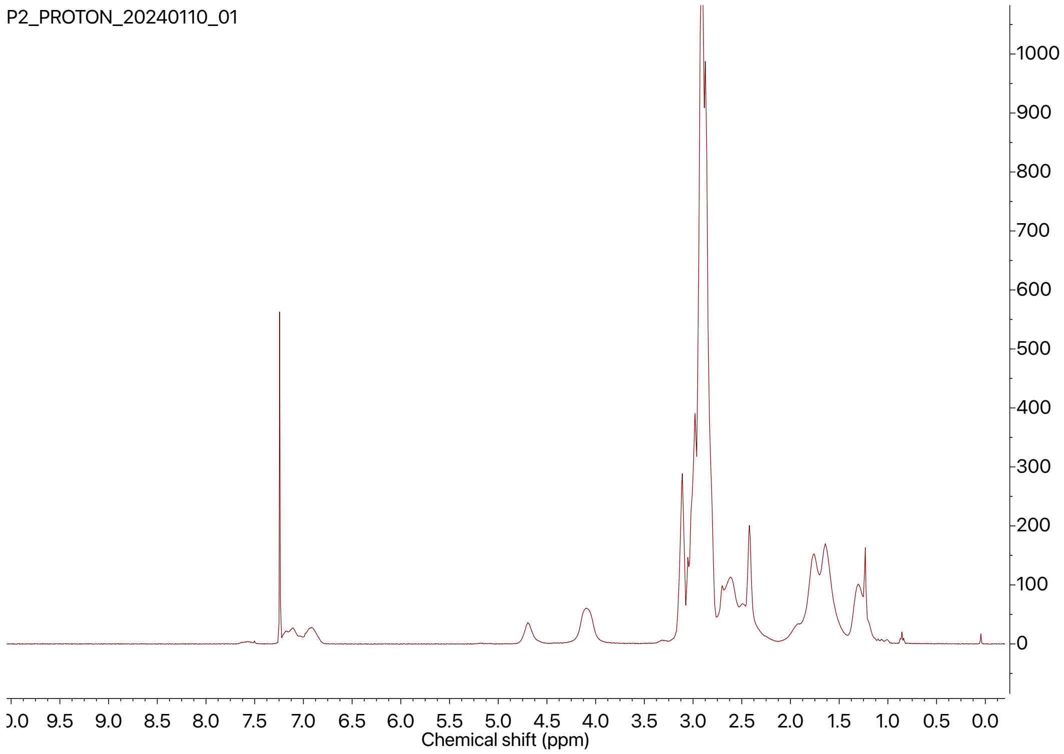

Figure S21. ^{1}H NMR spectrum of P2

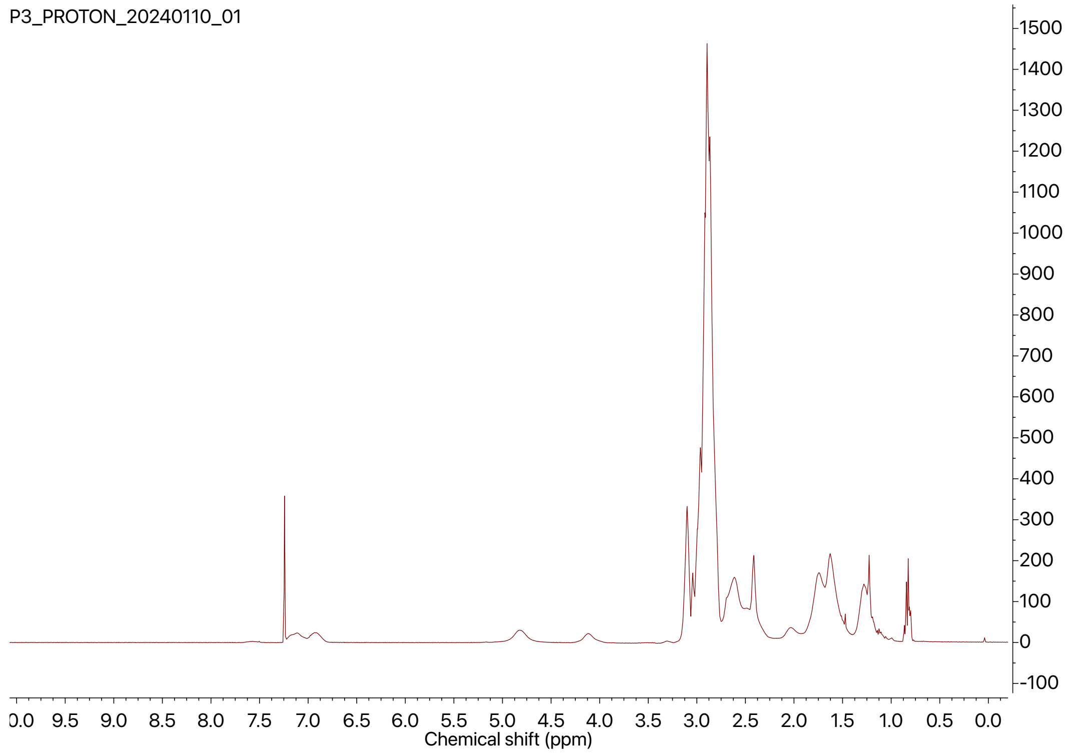

Figure S22. ^{1}H NMR spectrum of P3

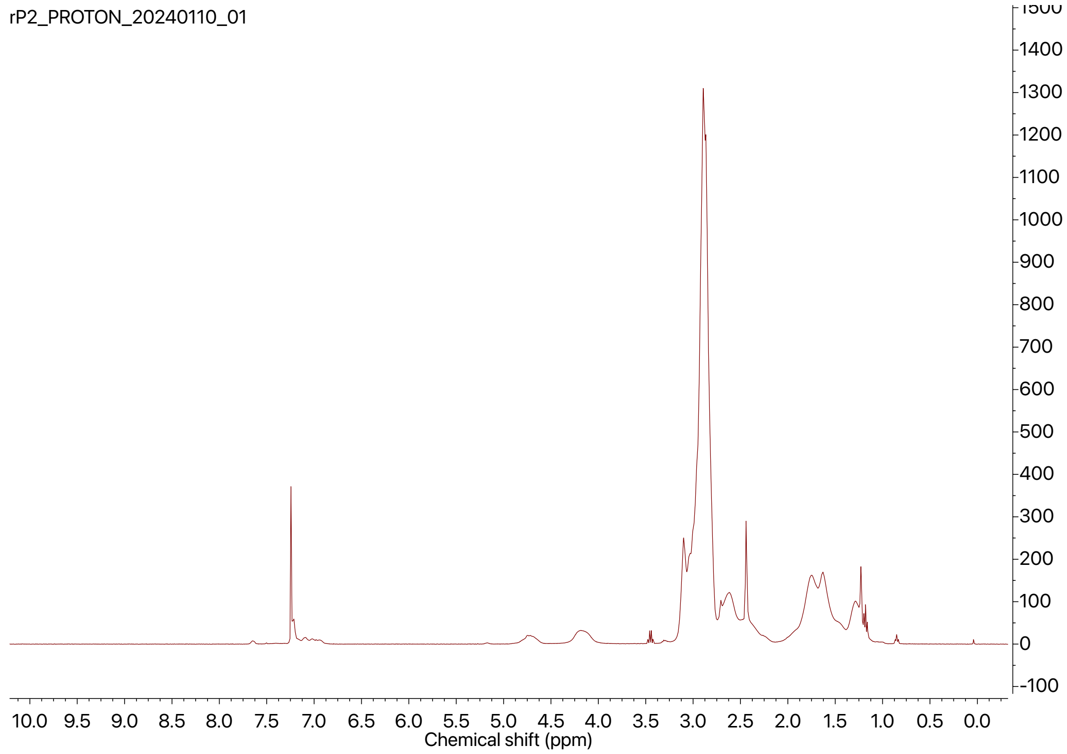

Figure S23. ^{1}H NMR spectrum of rP2

References

(1) Boutevin, B.; Rigal, G.; Rousseau, A.; Bosc, D. Synthese de materiaux polymers transparents

- Partie II Synthese et polymerisation d'acrylates et de methacrylates d'halogenoalkyle. *J. Fluor. Chem.* **1988**, *38* (1), 47-73.
(2) Imai, T.; Nishida, S.; Tsuji, T. Ring Opening Alkylation of Cyclic Ethers with α-Halogenoalkyllithiums in the Presence of Boron Trifluoride–Diethyl Ether. *J. Chem. Soc., Chem. Commun.* **1994**, (20), 2353-2354.
(3) Schomaker, J. M.; Borhan, B. Total Synthesis of Haterumalides NA and NC via a Chromium-Mediated Macrocyclization. *J. Am. Chem. Soc.* **2008**, *130* (37), 12228-12229.
(4) Piergentili, I.; Bouwmans, P. R.; Reinalda, L.; Lewis, R. W.; Klemm, B.; Liu, H.; de Kruijff, R. M.; Denkova, A. G.; Eelkema, R. Thioanisole Ester Based Logic Gate Cascade to Control ROS-Triggered Micellar Degradation. *Polym. Chem.* **2022**, *13* (16), 2383-2390.
